# Gene content, phage cycle regulation model and prophage inactivation disclosed by prophage genomics in the *Helicobacter pylori* Genome Project

**DOI:** 10.1080/19490976.2024.2379440

**Published:** 2024-08-12

**Authors:** Filipa F. Vale, Richard J. Roberts, Ichizo Kobayashi, M. Constanza Camargo, Charles S. Rabkin

**Affiliations:** a BioISI – Instituto de Biossistemas e Ciências Integrativas, Faculdade de Ciências, Universidade de Lisboa, Lisbon, Portugal; b Research Institute for Medicines (iMed-ULisboa), Faculty of Pharmacy, Universidade de Lisboa, Lisbon, Portugal; c New England Biolabs, Ipswich, MA, USA; d Research Center for Micro-Nano Technology, Hosei University, Tokyo, Japan; e Department of Computational Biology and Medical Sciences, Graduate School of Frontier Sciences, University of Tokyo, Tokyo, Japan; f Institute of Medical Science, University of Tokyo, Tokyo, Japan; g Laboratory of Genome Informatics, National Institute for Basic Biology, Okazaki, Aichi, Japan; h Division of Cancer Epidemiology and Genetics, National Cancer Institute, Rockville, MD, USA

**Keywords:** *Hp*GP, *H. pylori*, prophage, mobile elements, genome rearrangement, phage cycle

## Abstract

Prophages can have major clinical implications through their ability to change pathogenic bacterial traits. There is limited understanding of the prophage role in ecological, evolutionary, adaptive processes and pathogenicity of *Helicobacter pylori*, a widespread bacterium causally associated with gastric cancer. Inferring the exact prophage genomic location and completeness requires complete genomes. The international *Helicobacter pylori* Genome Project (*Hp*GP) dataset comprises 1011 *H. pylori* complete clinical genomes enriched with epigenetic data. We thoroughly evaluated the *H. pylori* prophage genomic content in the *Hp*GP dataset. We investigated population evolutionary dynamics through phylogenetic and pangenome analyses. Additionally, we identified genome rearrangements and assessed the impact of prophage presence on bacterial gene disruption and methylome. We found that 29.5% (298) of the *Hp*GP genomes contain prophages, of which only 32.2% (96) were complete, minimizing the burden of prophage carriage. The prevalence of *H. pylori* prophage sequences was variable by geography and ancestry, but not by disease status of the human host. Prophage insertion occasionally results in gene disruption that can change the global bacterial epigenome. Gene function prediction allowed the development of the first model for lysogenic-lytic cycle regulation in *H. pylori*. We have disclosed new prophage inactivation mechanisms that appear to occur by genome rearrangement, merger with other mobile elements, and pseudogene accumulation. Our analysis provides a comprehensive framework for *H. pylori* prophage biological and genomics, offering insights into lysogeny regulation and bacterial adaptation to prophages.

## Introduction

*Helicobacter pylori* is a common bacterial pathogen infecting ~50% of the human population worldwide, which is etiologically associated with gastritis, peptic ulcer disease, gastric adenocarcinoma, and mucosa-associated lymphoid tissue lymphoma.^1,2^ *H. pylori* is characterized by wide genomic diversity, which contributes to its pathogenicity and to host adaptation.^3^ The genome diversity is brought about by high mutation rates and fine mutual homologous recombination. *H. pylori’s* discrete population structure, resulting from within-household transmission and persistent infection, has been used to trace human migration.^1,4,5^

The genomic diversity of *H. pylori* is extensible to its prophages.^6–8^ Bacteriophages (phages) are viruses classified into two main categories: lytic and temperate. Lytic phages enter in a productive lytic cycle upon infection, resulting in phage genome replication and packaging, leading to progeny phage release through bacterial lysis. Conversely, temperate phages occasionally initiate replication upon host entry, while often follow the lysogenic cycle integrating into the bacterial genome to become a prophage. The prophages are vertically inherited during cell division, where the lysogenic state is maintained by the repression of phage lytic genes.^9^ Prophages can alter bacterial phenotypes, namely by encoding virulence factors, auxiliary metabolic genes, antibiotic resistance, immune evasion genes, and more.^10^ Additionally, prophage integration near or into critical bacterial genes may regulate gene expression, functioning as an on/off switch known as active lysogeny.^9^ Most genome sequences available consist of multiple contigs and lack methylome data, hindering prophage analysis for completeness, module order, and insertion site.

Previous research found prophage genes in ~20% of *H. pylori* genomes, belonging to the *Schmidvirus* genus.^6,11^ These prophages show genome synteny, a structured population,^7,12^ and high recombination rate.^8^ Yet, a comprehensive analysis of global *H. pylori* prophages and a model for the lytic–lysogenic transition are lacking. Annotating bacteriophage genomes is challenging due to high gene diversity, especially for genes involved in prophage lysogeny regulation and subversion of host metabolism, which are prone to rapid evolution.^13^ Accordingly, the *H. pylori* prophage pangenome includes ~60% unknown function proteins,^8^ hindering our understanding of phage cycle regulation, and the phage-bacteria arms race.^14^

Prophages often undergo a complex decay, leading to incomplete or cryptic remnants in bacterial genomes, preventing the lytic cycle.^15^ This decay process involves modular exchanges, point mutations, phage genome rearrangements, inactivation by other mobile DNA elements, and DNA deletion.^16^ The integration of prophages into the bacterial chromosome releases phage DNA from the selective pressures affecting replicating phages, allowing for gradual decay and diversification. Within the chromosome, prophages can recombine with other genetic elements, resulting in the formation of new chimeric phage types. Even heavily deleted and unable to follow the lytic cycle, prophage remnants can still serve as phage gene reservoirs in the bacterial chromosome.^17^ Thus, cryptic prophages may benefit the host by providing host adaptation features.^18^ While *H. pylori* may contain cryptic prophages, their prevalence and decay processes remain poorly understood.

The *H. pylori* Genome Project (*Hp*GP) gathered 1011 genomes of clinical isolates from 50 countries. Here, we analyzed this high-quality dataset to assess prophage occurrence for the first time in a large collection of complete, closed genomes, enriched with methylome data for comprehensive prophage genomics. Prophage sequences are often scattered across the genome and can impact the methylome. We suggest that the bacterial genome shuffling, merging of prophages with other mobile elements, and pseudogene accumulation constitute the *H. pylori* prophage decay process. This unprecedented collection of curated prophages provides a resource to study phage cycle regulation and prophage inactivation, opening the way for further insights through wet-lab experiments.

## Results and discussion

### *Prophages are relatively common elements in* H. pylori *genomes*

Of the 1011 *Hp*GP genomes, 1004 are closed complete genomes and seven could not be circularized. All identified prophage sequences were manually inspected to accurately define and delimit each sequence. Most publicly available genomes correspond to contigs/scaffolds (e.g., 5393/6301; 83.6% fragmented genomes at NCBI). Using closed genomes is of extreme importance as it enables us to determine whether the prophage sequences are spread across the bacterial chromosome or lie adjacent to each other and in what order.

Out of the *Hp*GP set, 368 prophage sequences were identified in 298 genomes (29.5%, 298/1011). PhiSpy algorithm^19^ did not detect any additional novel prophage in addition to BLASTn and phage word search. Among the genomes with prophages, 96 (32.2%, 96/298) carried presumably complete full-length prophages. Out of the total prophage sequences found (368), the majority (272) were incomplete and undergoing a decay process, which potentially reduces the prophage carriage burden. However, genes from remnant prophages might reintegrate into the bacteriophage gene pool through recombination with other infecting phages.^20^ Prophage regions that were uninterrupted and spanned over 20 kb, featuring a composition of prophage genes that defined the boundaries of the sequence, were marked as complete prophages. In contrast, prophage regions that were shorter in length, lacked phage genes to define their boundaries, or instances where most genes were not phage genes, were categorized as incomplete. Prophage frequency depends on the restriction and modification (RM) systems present.^21^ The extreme diversity in the *H. pylori* methylome^22^ suggests that these RM systems protect the host from phage infection, justifying the moderate prophage frequency, and specific prophage populations (discussed below) across different *H. pylori* populations.

In the *Hp*GP dataset, complete prophages comprised, on average, 1.7% of the bacterial genome size, while incomplete or remnant prophages constituted, on average, 0.9% of the genome size. Related to the gene content, complete prophages encode an average of 2.1% of the total *H. pylori* genes, while the remnant prophages encode, on average, 1.1% of the total genes. Accordingly, *H. pylori* genomes with prophages are significantly larger than the ones without them (bacteria with prophage genome size average was 1,652,199 ± 35248 bp, and without was 1,626,977 ± 40819 bp, Mann-Whitney U-test p-value <.0001), with a tendency toward lower guanine and cytosine percent (GC%) (bacteria with prophage GC% average was 38.91 ± 0.14%, and without was 38.93 ± 0.15%, t-student test p-value = .0153) (Supplementary Figure S1.a.). Prophage genomes have a GC% content lower than the bacterial one (36.4% vs. 38.9%), suggesting horizontal gene transfer.^23^ Complete prophages have, on average, 28.5 Kb; standard deviation (SD), 6.9 Kb, while the incomplete ones have 10.6; SD, 7.2 Kb (Supplementary Figure S1.b.).

### *Prophages present an uneven prevalence by country of isolation and* H. pylori *ancestry*

The prevalence of prophages varies geographically (Fisher's exact test p-value <.0001; Supplementary Figure S1.c., Table S1). The highest (70%; 7/10) prevalence was observed on Russian genomes, while the absence of prophages was noted in genomes from Canada (*n* = 20), Gambia (*n* = 5), Guatemala (*n* = 3), and Kazakhstan (*n* = 2). Nevertheless, the small sample size in some countries may limit the accuracy of these estimates. An interesting observation is the absence of complete prophages among the Japanese genomes. It is worth noting that *H. pylori* phages identified in Japanese strains, such as KHP30 and KHP40, were reported to exist as extrachromosomal episomes, indicative of a pseudolysogenic state.^24^ However, we could not identify the presence of such phage episomes in the *Hp*GP dataset, neither for Japan nor for other genomes. It is possible that the analyzed Japanese strains could be susceptible to infection by KHP30-like phages, which appear to be present as an episome despite carrying an integrase gene.

**Figure 1.**
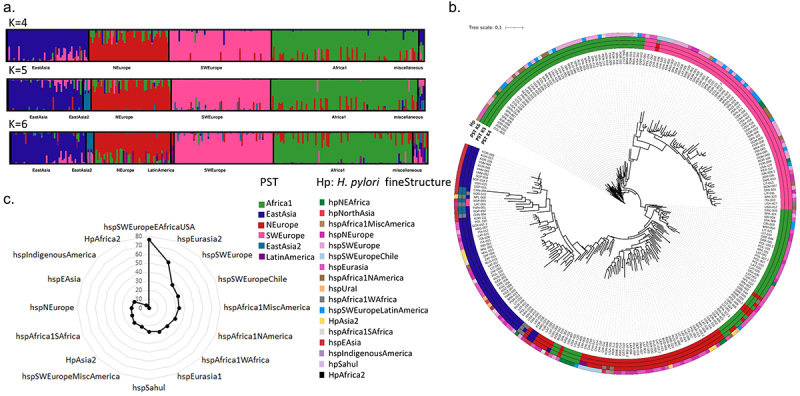
Global overview of *H. pylori* prophage abundance and population structure.

The significantly uneven prevalence of prophages is also exhibited by ancestry (Fisher's exact test p-value = <.0001). *H. pylori* populations like hspSWEuropeEAfricaUSA (76%; 13/17) and hspEurasia2 (55%; 43/78) have the highest prevalence of prophages, while hspIndigenousAmerica (1 out of 22) and hpAfrica2 (0 out of 4) have the lowest (Table 1, Figure 1c). The variable prevalence mirrors the evolutionary trajectory of phage acquisition and loss, along with local adaptation. One possible explanation is that the *H. pylori* populations without prophages diverged before prophage acquisition or originated from an ancestral subset without prophages. If the absence of prophages in a larger sample is confirmed, then this could explain why the hpAfrica2 population, the most divergent among *H. pylori* populations, lacks any prophage, possibly indicating a split from other *H. pylori* populations prior to prophage acquisition. Thus, the limited number of hpAfrica2 genomes in our *Hp*GP dataset underscores the importance of investigating prophage presence in more samples from this group. The distinct prevalence across populations suggests that prophages could contribute to the evolution of more adapted *H. pylori* subpopulations. Some bacterial populations may have acquired mechanisms to regulate or control prophage induction and maintain a balance between lysogeny and the lytic cycle. This equilibrium could result in more stable prophage integration and a higher abundance of prophages in those populations.

(a) Prophage sequence typing (PST) based on the integrase and holin genes. The DISTRUCT plot of the Bayesian population was assigned using STRUCTURE and an admixture model for K populations (K = 4 to K = 6) for 260 genomes containing the prophage integrase and holin genes. Each prophage is represented by a vertical line divided into K colored segments representing the membership coefficients in each cluster. (b) Concatenated integrase and holin phylogenetic tree. Outer circles represent population membership for PST with 4, 5, and 6 populations assigned, and for the *H. pylori* fineSTRUCTURE assigned population. (c) Uneven prevalence of prophage elements by *H. pylori* ancestry (*p* < .0001). The percentage of prophage presence by *H. pylori* fineSTRUCTURE ancestry is indicated by a black line.

**Table 1.** Prophage prevalence by *H. pylori* ancestry.

*H. pylori* population	Total number of genomes	Genomes with prophage (n)	Genomes with prophages (%)	Genomes with complete prophages (n)	Genomes with complete prophages (%)	Proportion of complete prophages (%)
hspSWEuropeEAfricaUSA	17	13	76.5	0	0.	0.
hspEurasia2	78	43	55.1	13	16.7	30.2
hspSWEurope	175	64	36.6	24	13.7	37.5
hspSWEuropeChile	43	15	34.9	3	7.	20.
hspAfrica1MiscAmerica	21	7	33.3	1	5.	14.
hspAfrica1NAmerica	57	18	31.6	6	11.	33.
hspAfrica1WAfrica	7	2	29.	0	0.	0.
hspEurasia1	173	50	28.9	21	12.1	42.0
hspSahul	15	4	26.7	2	13.	50.
hspSWEuropeMiscAmerica	111	25	22.5	5	5.	20.
HpAsia2	34	8	23.5	5	15.	63.
hspAfrica1SAfrica	38	8	21.1	5	13.	63.
hspNEurope	50	10	20.0	0	0.	0.
hspEAsia	166	30	18.1	11	6.6	36.7
hspIndigenousAmerica	22	1	4.6	0	0.	0.
HpAfrica2	4	0	0.	0	0.	0.
Total	1011	298	29.5	96	9.5	32.2

Incomplete (or remnant) prophages are the majority (71.5%; 213/298) in *H. pylori* genomes. Out of the 213 genomes containing remnant prophages, 202 have incomplete prophages, while 11 have both complete and incomplete prophages.

The prophage prevalence was similar regardless of disease status of the host (i.e., non-atrophic gastritis, advanced intestinal metaplasia and gastric cancer, Chi-square test p-value = .4254), suggesting that prophage carriage may not have a direct impact on disease outcomes. Furthermore, the occurrence of prophages is not associated with the virulence factor CagA, as annotated by NCBI (Chi-square test p-value = .4254). This suggests that prophage carriage may not have a direct impact on disease outcomes.

### H. pylori *prophages have a structured population*

We previously used prophage sequence typing (PST) based on integrase and holin genes to categorize five phage populations: Africa1, EastAsia, SWEurope, NEurope, and Colombia.^6,12,25^ Despite utilizing just two phage genes, this approach holds importance due to the common incompleteness of prophage sequences. The higher prevalence of these two genes compared to complete genomes makes the method valuable. PST evaluated 260 prophage sequences, surpassing the 96 complete prophages, enabling the analysis of more prophage sequences. STRUCTURE analysis offers the best K in between 4 and 6 prophage populations. At K = 5, a new EastAsia2 population emerges, encompassing genomes from China, Korea, Singapore, and Taiwan (Supplementary Table S2). At K = 6, the former Colombia population clustered with other prophages from Latin America, leading to a proposed name change to Latin America (Figure 1a). The phylogenetic tree (Figure 1b) based on concatenated integrase and holin genes shows prophage sequences clustering by their PST population. Interestingly, specific *H. pylori* ancestries show nonrandom prophage populations (Figure 1b; Supplementary Figure S2c), corroborating a previous finding.^26^ For instance, the *H. pylori* subpopulation hspEAsia is mainly lysogenic for EastAsia and EastAsia2 prophage populations, while the only *H. pylori* lysogen from subpopulation hspIndigenousAmerica carries a prophage with NEurope ancestry (Supplementary Figure S2c). This suggests that the introduction of prophages into the hspIndigenousAmerica population (typically found in Indigenous populations) arose after the introduction of *H. pylori* European lineages, reflecting the European colonization of America. Similarly, when analyzing by country of isolation, the same tendency is observable. For instance, Peruvian genomes are lysogens of prophages with ancestries from SWEurope, NEurope and LatinAmerica (Supplementary Figure S2b). No relationship is observable between prophage ancestry and associated disease (Supplementary Figure S2a). These observations point to prophages being more involved in *H. pylori* adaptation to certain geographies, rather than being associated with disease outcome. Each *H. pylori* subpopulation has its own prophage circulating populations (Supplementary Table S2), and this feature may confer niche-specific adaptation.

### Recombination between prophage populations takes place on intra- and inter-population scales

**Figure 2.**
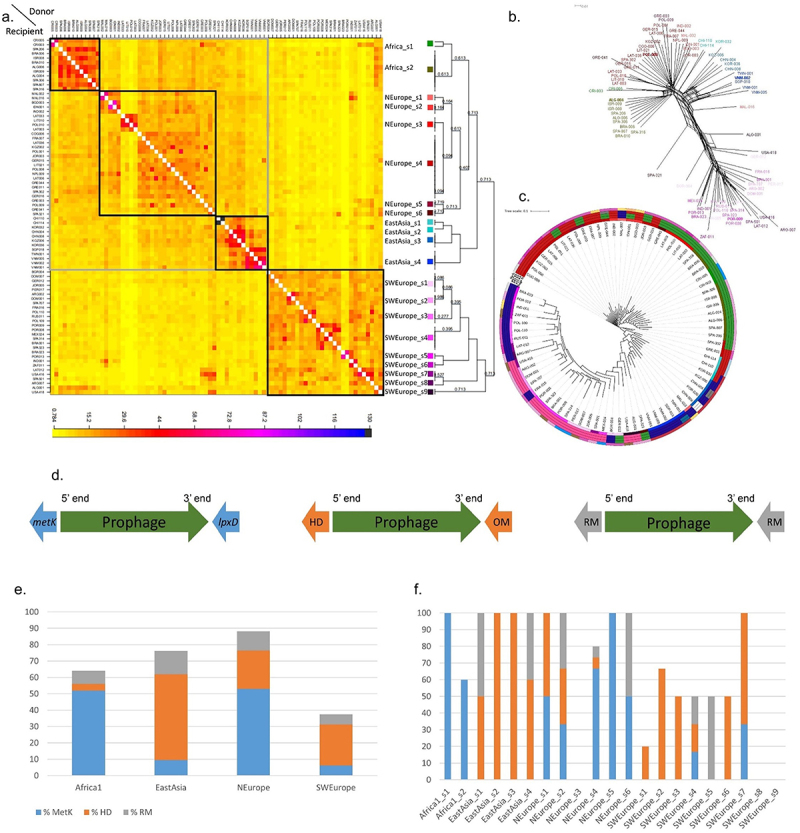
Population structure and phylogenetics of the complete *H. pylori* prophages.

The recombination among prophages provides an opportunity to study their diversity and gene flow and to disclose aspects related to human migrations. We have applied ChromoPainter/fineSTRUCTURE to study the population genetic structure, admixture and recombination across the complete prophages (80 genomes) using genome-wide single nucleotide polymorphism (SNP) data. We have excluded identical prophage sequences (GRE-041 and GRE-046 have highly similar bacterial genomes pointing to the possibility of a strain infecting distinct Greek patients), as well as prophages presenting large genome inversions and cargo genes, since these pose an obstacle to multiple genome alignment. This is observable when contrasting the networks (Supplementary Figure S3a and Figure 2b) obtained with 95 or 80 complete prophages, in which the former shows a rather box-like network, indicating incompatibilities in the data that cannot be explained by a simple tree-like evolution scenario. Also the phylogenetic trees for 95 and 80 complete prophage genomes (Supplementary Figure S3C and Figure 2c) evidence longer branches for prophages carrying inversions and cargo genes, reporting more genetic change or divergence, which, in fact, is attributed to the prophage genome rearrangement or cargo gene presence rather than to a true divergence of the DNA sequence. Additionally, the gene cluster comparison of all prophage regions (Supplementary Figure S4) also allows the detection of rearrangements and cargo genes.

(a) Co-ancestry matrix of *H. pylori* prophage genomes. The color of each matrix square represents the expected number of fragments exported from a donor genome (column) to a recipient genome (row), inferred by Bayesian clustering in fineSTRUCTURE. The black and gray boxes indicate evidence of higher recombination between genomes based on the co-ancestry matrix. (b) SplitsTree phylogenetic network reconstructed using the complete genome alignment of 80 complete prophages. Prophage genomes in bold are reference prophage representative of the main populations. Tip colors correspond to those of Figure 2a. (c) Maximum-likelihood phylogenetic trees from complete prophage genome alignment. Circles from inside to outside: phage fs – prophage population determined by fineSTRUCTURE coded as in Figure 2a; PST K4, PST K5 and PST K6 – populations determined by phage sequencing typing (based on integrase and holin) coded as in Figure 1a; Hp fs - bacterial population determined by fineSTRUCTURE. (d) Schematic map of the prophage insertion sites. Prophage sequence is in green evidencing its 5’ end (left end) and 3’ end (right end). The most common insertion site is found from left to right between the bacterial genes: *metK* and *lpxD* (genes in blue); HD domain and outer membrane protein (genes in orange); and between RM genes (genes in gray). (e) Percentage of prophage insertion sites by PST K = 4, according to site: S-adenosylmethionine synthetase *metK* gene at 5’ end (%metK), HD family hydrolase gene at 5’ end (%HD), and flanked by restriction and modification (RM) genes (%RM). (f) Percentage of prophage insertion sites as in Figure 2d by fineSTRUCTURE population.

The co-ancestry heatmap (Figure 2a) with unevenly distributed colors shows variation in the number of DNA fragments inferred to be donated by other donor individuals. The principal fineSTRUCTURE split shows SWEurope phage population genetic differentiation (Figure 2a), which is also supported by the network (Figure 2b) and phylogenetic tree (Figure 2c) analyses. This observation suggests genetic isolation, also supported by long branches in the network and phylogenetic tree, confirming previous findings.^8^ In the principal component analysis (PCA), whose first six components explain 44.7% of the variance, SWEurope prophages diverge from others along the first component (Supplementary Figure S3b). Accordingly, the nine sub-groups found within the SWEurope (SWEurope_s1 to SWEurope_s9) population mainly exchange DNA segments among themselves. The other three populations Africa, NEurope and EastAsia present 2, 6, and 4 sub-groups, respectively. These three populations are mainly receivers of DNA segments from individuals of the same population. Still, they also receive DNA segments from individuals of these three populations (i.e., they have signatures of inter-population recombination). It is noteworthy that none of the prophages assigned to LatinAmerica population by STRUCTURE were included in the fineSTRUCTURE analysis, while one prophage from the EastAsia2 population assigned by STRUCTURE (from Taiwan, Supplementary Table S2) corresponds to fineSTRUCTURE EastAsia_s4, the most distant among the EastAsia subgroups. The four main prophage populations also revealed a high degree of differentiation among populations and genetic diversity within populations. Globally, PST, fineSTRUCTURE and PCA revealed a clear prophage population structure. The fine-scale genetic structure detected by haplotype sharing (fineSTRUCTURE) explained substantially more variance in complete prophages than PCA and PST. The recombination signatures found provide prophages with increased diversity, which may help them escape bacterial immunity by recombining with other phages or prophages, and adaptation to new environments/bacterial populations.

Looking at particular prophage subpopulations it was possible to establish links with the human migrations, specially by joining data from isolate country of origin, *H. pylori* ancestry and phage fineSTRUCTURE (Supplementary Table S2). The subgroups SWEurope_s9 (from Algeria and the US) and Africa_s2 (from Algeria, Brazil, Israel, and Spain) exchange DNA chunks between them, meaning that these two distinct prophage populations recombine in Algeria, pointing to the migratory history between the North African region and the Iberian Peninsula, while all isolates from Algeria shared the same bacterial ancestry. American strains often exhibit a mixed population composition resulting from European colonization, the historical practice of slavery, and frequent migratory movements. A noteworthy observation pertains to the apparent isolation of the SWEurope_s9 subgroup (from Portugal and Brazil), which reflects the shared historical bond between the two countries attributed to the former’s colonization. Concerning the East Asian subgroups (Supplementary Table S2), it is worth noting that the subgroups from Southeast Asia (EastAsia_s4: Singapore, Taiwan and Vietnam) and the Far East (EastAsia_s3: China, Kyrgyzstan, and Korea) exhibit more pronounced intra-group recombination in line with their specific geographical locations, to a lesser extent with each other, and distinctly separated from the two other East Asian groups, like EastAsia_s1 from Chile. Regarding NEurope subgroups, s1 (Malaysia) and s2 (Bangladesh, Indonesia, India) extendedly recombine among each other and belong to more close geographic locations. NEurope_s3 essentially recombine among themselves, corresponding to Baltic countries Latvia and Lithuania, and neighboring Poland. The remaining subgroups within NEurope recombine among them stemming from diverse sources, primarily comprising European nations. Finally, Africa_s1 comprises prophages from Costa Rica, and Africa_s2 (Algeria, Brazil, Israel, and Spain) that recombine more among each subgroup. Only a limited number of African prophages are available for tracing the spread of prophages and migrations within this continent.

### H. pylori *prophages have hotspots for integration sites and occasionally disrupt bacterial genes*

*H. pylori* prophages do present hotspots for integration that are related to the main populations (Figure 2d-f, Supplementary Table S2). The most prevalent insertion spot, found in 27.9% (83/298) of genomes with prophages, is between the methionine adenosyltransferase (*metK*) and UDP-3-O-(3-hydroxymyristoyl)glucosamine N-acyltransferase (*lpxD*) genes, particularly abundant in Africa1 and NEurope prophages (Figure 2d), consistent with a previous study.^7^ When considering only the group of complete prophages analysed by fineSTRUCTURE, there is a confirmation that the subpopulations belonging to Africa1 are exclusively integrated between these two genes, while the subpopulations from NEurope are predominantly integrated here (Figure 2e). The proteins encoded by these genes catalyze the formation of S-adenosylmethionine (AdoMet) from methionine and ATP (MetK) and are involved in the biosynthesis of lipid A, a phosphorylated glycolipid that anchors the lipopolysaccharide to the outer membrane of the cell (LpxD).

The gene encoding an HD family hydrolase, found in 25.8% (77/298) of the genomes, is the second most common hotspot for prophage insertion, situated at the 5’ end of the prophage (left end of the prophage), with a higher frequency in EastAsia and SWEurope prophages (Figure 2e). When considering only complete prophages, the subpopulations determined by fineSTRUCTURE that are most prevalently inserted here are also from SWEurope and EastAsia (Figure 2f). The bacterial gene found at the 3’ end of the prophage (right end of the prophage) is not conserved for this hotspot. However, although not entirely conserved, the bacterial gene found on the 3’ end of the prophage most frequently present (48 genomes) codes for an outer membrane protein (*homA*, according to Prokka annotation), and the second most frequent (nine genomes) a gene coding for S-adenosyl-L-methionine hydroxide adenosyltransferase family protein.

Another remarkable and novel observation is that prophages often insert adjacent to RM systems (8.1%, 24/298) (Supplementary Table S3). An RM system, a prokaryotic immune system, consists of a DNA methyltransferase, which transfers a methyl group to a specific DNA sequence, and a restriction enzyme, which cleaves DNA lacking that methylation.^27^ However, these systems are often distinct among genomes, and in these cases, the insertion site is not truly a unique site between the bacterial genomes. We could verify that three of these RM systems appear repeated as insertion sites for prophages.

The conservation of the integration site suggests a specificity of the phage integrase for DNA sequences found at these hotspots. Indeed, the phylogenetic analysis and population structure support the existence of distinct populations of prophages that have characteristic insertion sites, which is also observable for the integrase gene.^11^ The conservation of the integration site may also reflect prophages being in decay and the presence of vertical transmission of prophages within each *H. pylori* lineage. This scenario could apply particularly to remnant prophages, whose incompleteness hampers phage excision. Inspection of the intergenic regions flanking complete prophages revealed conserved sequences at each integration hotspot, which may constitute the integrase recognition sequences. The intergenic region between bacterial and prophage for complete prophages integrating between bacterial genes *metK* and *lpxD* has, on average, 297 bp (SD, 3 bp). The intergenic region at 5’ end (between *metK* and first prophage gene) displays a notably conserved sequence, characterized by two specific conserved segments: TAAGCTATAATAAGCC (at −56 bp from prophage start) and GATTATTTTAATAAGGACAA (at −24 bp from prophage start), that may be important for integrase recognition and prophage insertion. The intergenic region at the prophage 3’ end (between the last prophage gene and the bacterial *lxpD* gene) has, on average, 668 bp (SD, 150 bp) and is characterized by repeat sequences (average size of 42 bp, SD of 73 bp) that may result from prophage insertion. Similarly, the intergenic region of the second most common insertion site between HD domain and an outer membrane/hypothetical protein, with 399 ± 57 bp, has three conserved regions upstream prophage start: TTTTTAAATAAAA (at −167 bp from prophage start), TTTTTTTCGTATAATAA (at −138 bp) and CAAAATAGTATAAA (at −24 bp). The intergenic region downstream of the prophage is characterized by shorter sequence repeats, with an average of 10 bp (SD, 32 bp). These repeats may be generated after the integration of prophage into the host genome via recombination.^23^

Taking into consideration the insertion sites and the co-ancestry heatmap (Figure 2), there appear to have occurred two hypothetical major prophage prototypes, each with its specific integration site (one integrating adjacent to *metK* and the other to HD domain) that have recombined, giving rise to the other populations. Two hypotheses arise, introducing uncertainty: either SWEurope or NEurope recombined, potentially resulting in EastAsia and Africa1, or an alternative scenario involving a recombination between NEurope and EastAsia, potentially giving rise to SWEurope. The recombination opportunity may have arisen from simultaneous infection of the same bacterial genome by these prototypes; or by the presence of mixed *H. pylori* infections of the human host. Giving strength to the former, we found two distinct genomes (LAT-012 and NPL-009) where a complete and a remnant prophage appear at these two conserved insertion sites, proving an opportunity for recombination. In the case of NPL-009, both prophages belong to the NEurope population and are identical in their sequences. However, LAT-012 prophages belong to distinct prophage populations; the complete prophage is inserted adjacent to the HD domain gene within the SWEurope_s7 population, whereas the remnant belongs to NEurope and is inserted adjacent to the *metK* gene. This LAT-012 remnant prophage has signs of multiple rearrangements dispersed across two bacterial genome sites. One segment of the remnant prophage is adjacent at its 5’ end to the *metK* gene and merges at its 3’ end with a *tfs* region. Meanwhile, the other segment of the remnant prophage is positioned approximately 650 kilobases apart, next to the *lpxD* gene.

Gene disruption upon insertion is among the factors that render prophages non-neutral to bacteria. Disrupted genes are detectable adjacent to 14.8% (109/[96 + 272]x2; among the 736 genes flanking prophages, 109 are pseudogenes) of all *H. pylori* prophage insertion sites (Supplementary Table S4). Whether prophage excision leads to gene integrity restoration remains to be determined. Most disrupted genes are hypothetical proteins, followed by RM genes, a gene class known to be variable among *H. pylori* genomes.^22^ Noteworthy, among the examined bacterial genomes, the *H. pylori* genome carries the largest number of RM genes.^28^ The repertoire of RM systems and other sequence-specific DNA methyltransferases in *H. pylori* is strain-specific and variable, forming a unique and ever-changing methylome.^22,29,30^ *H. pylori* provided a unique opportunity to understand the interaction between these epigenetic systems and prophages. Indeed, 62.5% (15/24) of the prophage genomes flanked by RM genes present RM disruption, disrupting bacterial genomic methylation (Supplementary Table S3). Disrupting methyltransferase genes altered the phenotype, evaluated by the epigenomic methylation found in the genome (i.e., the methyltransferase recognition target site was not methylated). Whenever a methyltransferase gene was disrupted by prophage insertion, the methyltransferase recognition target site was not methylated (genomes GER-001, IDN-001, ITA-026, LIT-041, POL-007, POL-102, POL-103, POL-104, POR-013, SGP-018, SPA-301, SPA-709, SPA-801, USA-427), unless a second methyltransferase recognizing the same target site is present (genomes DOM-009, GRE-041, SPA-323, USA-422, USA-434 and VNM-002, in Supplementary Table S3). Concerning the disruption of the companion endonuclease alone, in half of the cases the methylation is present (DOM-009, IDN-001 and USA-422) and absent in the other half (BGR-007, POL-007, and USA-429). Of note, not for all cases of RM system disruption it is possible to verify if there is a phenotypic alteration, like when the target methylation site is undetermined or when there are constraints related to decoding 5-methylcytosine using PacBio sequencing. The RM system disruption by prophages suggests a mechanism to counteract host immunity, and another form of interplay between mobile genetic elements, where prophages are acting as epigenetic switches. Moreover, the disrupted gene list also reveals a set of genes whose loss of function is not essential for cell survival. Here, we have verified that prophage integration can disrupt host genes, but prophage excision may eventually reverse this disruption.^31^

### H. pylori *complete and remnant prophage present genome synteny*

The synteny of gene cassettes is often conserved between different phages, especially among bacteriophages of small genome size, presumably representing a feature of bacteriophage evolution.^32^ Both complete and incomplete prophages presented a well-conserved pattern of genomic organization and synteny (Supplementary Figure S4). In terms of gene organization throughout the prophage genome, gene order is kept among prophage even across distinct populations, pointing to i) distinct prophage populations sharing a common evolutionary lineage; ii) these genes being essential for phage function and being conserved over time; iii) for the importance of the gene order in regulation; and iv) its role in governing the phage life cycle.

### H. pylori *genome rearrangement split prophage sequences leading to prophage remnants*

An open question regarding *H. pylori* prophages concerns the existence of polylysogeny (i.e., carrying multiple prophages). Prophage fragmentation makes it challenging to determine the existence of polylysogeny. We found 20.5% (59/298) of *Hp*GP genomes harboring multiple prophages, considering both complete and incomplete. Of these 59 genomes, 49 had two regions, nine had three, and one had four. In most (49) genomes, these prophage regions correspond to prophage fragmentation) by the bacterial chromosome, rather than the presence of distinct prophages. In 15 genomes, however, there is indeed more than one prophage present.

**Figure 3.**
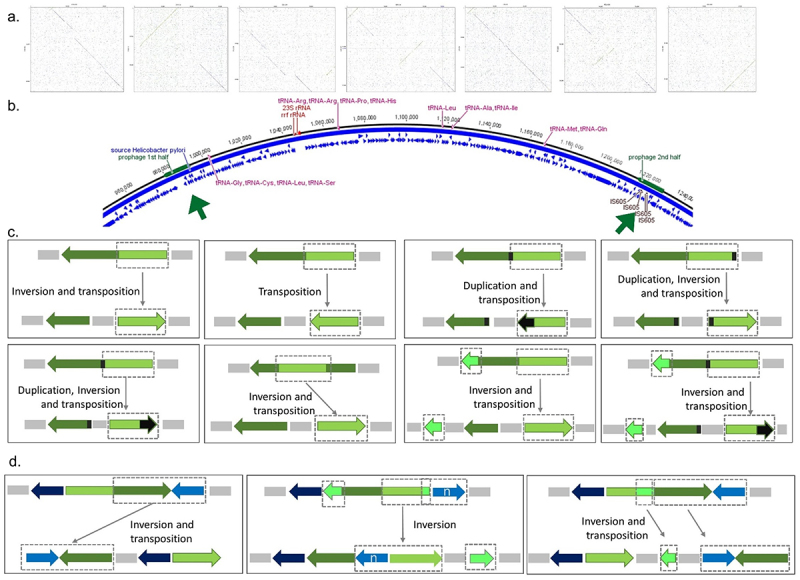
Prophage inactivation by genome rearrangement in 298 complete genomes.

Noteworthy, in five of these 15 genomes (LAT-012; POL-007, SPA-322, LAT-036; POL-009), there is both a prophage genome split and more than one prophage. In the case of prophage fragmentation, the regions of the prophage appear scattered by the bacterial genome, whether all prophage pieces are still present. Often not all pieces that make a complete prophage appear to be present. We hypothesize that the rearrangement by inversion within the prophage genome (Figure 3a), or by the *H. pylori* genome rearrangement involving prophage regions (Figure 3b) leads to prophage remnants. The rearrangements involve inversions, transpositions, and duplications (Figure 3b,c). These genome rearrangements involve dispersing just the prophage regions (Figure 3b), or prophage regions and bacterial regions that are usually present in synteny blocks (Figure 3d). Prophage inactivation by prophage genome rearrangement may constitute a new strategy of host defense leading to prophage decay, where the rearrangements pulverize prophage segments across the genome, and later, some may be lost. Indeed, in most genomes we observe prophage remnants (213/298). Of these, 11 genomes have completed and other fragmented prophages, and 202 have only incomplete prophages.

(a) Prophage genome inversion (present in 13; 4.4%) as shown by dotplots of 7 complete prophages with inversions, depicted as green lines in perpendicular directions. (b) Prophage split into two fragments across the bacterial genome, represented as green boxes and arrows (present in 49; 16.4%). (c) Schematic representation of prophage genome rearrangement involving inversion, transposition and/or duplication (present in 32; 10.7%). (d) Schematic representation of the case of bacterium and prophage genome rearrangement implying inversion and/or transposition (present in 17; 5.7%).

Interestingly, prophages often break in two halves with the breaking point being in a prophage gene with high homology to bacterial genes (DUF3519 domain, DNA binding chromosome segregation), pointing to a genome rearrangement generated by homologous recombination involving DNA sequences with near identical sequences, one bacterial and the other from a prophage.

Concerning the 15 polylysogenic genomes, in six there is a (partial) prophage duplication, meaning that only nine cases had vestiges of polylysogeny. In all polylysogenic genomes, the combinations found were complete and remnant(s) prophages, or only remnant prophages, with dot plots showing the absence of sequence homology (genomes GER-015, LAT-012, ISR-009, POL-007, SPA-322, SPA-323, POL-103, COL-301, and SPA-707), pointing to distinct prophages. In none of the cases, two distinct complete prophages were found, pointing to prophage mediated superinfection exclusion, either by prophage insertion site occupancy, or the presence of a prophage regulator preventing infection by the same phage. There are two hypothetical prophage repressor genes (see below) commonly present in complete prophages, which may be associated with superinfection exclusion. Interestingly, when there is more than one distinct prophage per genome, in no case were the two repressors observed in all the prophages present (i.e., at least one of the prophages did not have these repressors). Our data indicate the absence of true polylysogeny in the *H. pylori* genomes. Small bacterial genomes generally have a low co-occurrence of prophages,^33^ as is the case of *H. pylori*.

### Prophage can merge with other mobile elements

Interestingly, we often observed the merging of prophage sequences with other mobile elements (Supplementary Table S5). The most common pattern involved insertion sequences, found integrated in the prophage in 48% (145/298) of the genomes. Insertion sequences may disrupt the gene regulation within the prophage sequence, impacting the phage life cycle. Additionally, prophages merged with a Type IV secretion (*tfs*) cluster were found in 10% (34/298) and, less frequently, with *cag* pathogenicity island (PAI) in 3.4% (10/298) of the genomes. The convergence of mobile elements may disrupt *cag*PAI and *tfs* by prophages, and vice-versa. Typically, prophages were found adjacent to *cag4* or *cagS*. The former is known to be a splitting point of *cag*PAI into a right segment (*cagI*) and a left segment (*cagII*), usually split by insertion sequences, which provide intermediate pathogenicity phenotypes.^34^ Here, we show for the first time that prophages can also split *cag*PAI. Prophages may contribute as well to remnant *tfs* plasticity zone, a region that is important for the colonization of *H. pylori* and disease progression.^35^ When merged, mobile elements lose their typical gene order, and can together be vertically transmitted by cell division, gained and/or lost, as well as to impose intermediate phenotypes, regarding the pathogenicity of the strain. Merging with other mobile elements changes the genomic organization of prophages, probably disrupting their gene regulation. This may constitute a weapon in the phage-bacteria arms-race.

### Remnant prophages contribute to the expansion of the pangenome and exhibit greater levels of pseudogenization

**Figure 4.**
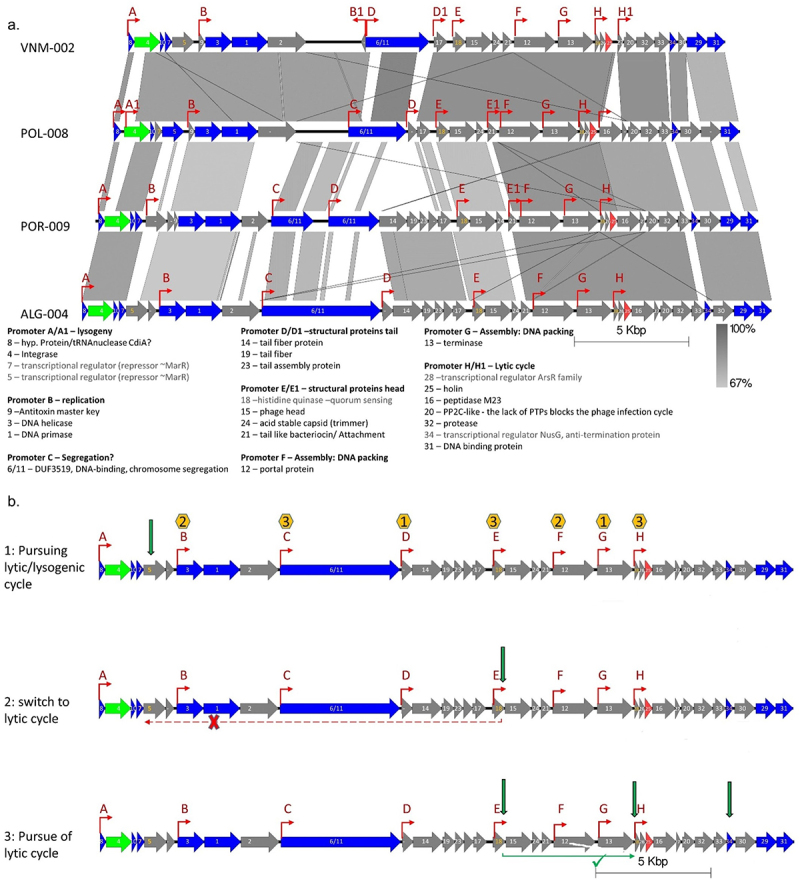
Proposed mechanism for lysis/lytic cycle regulation in *H. pylori* prophages.

Considering complete and incomplete prophage regions, the phage pangenome comprises 147 genes (Supplementary Table S6), including cargo genes (see below). Out of these, 57 (38.8%, 57/147) are hypothetical proteins, adding complexity to *H. pylori* bacteriophage biology understanding. The high genetic diversity within *H. pylori* prophages is reflected in the proportion of genes with unknown functions. Within the pangenome, some genes are shared with integrative elements, like type IV secretion proteins, that may have arisen from merging distinct mobile elements (see above). None of the genes is shared between all prophages, which was expected since remnant prophages were included. Focusing solely on the complete prophage group, we identified a pangenome of 107 genes. Among these, nine genes were present in at least 90% of the prophages (Figure 4a), with a decreasing proportion of hypothetical proteins (28.0%, 30/107). We cannot disregard that the high genetic diversity of sequences observed may contribute to identifying them as different genes, even if they code for proteins with a similar function, as is observed for insertion sequences within prophages.

(a) Gene cluster of complete prophage references representative of the four main prophage populations (bold tips in Figure 2b), VNM-002 – EastAsia, POL-008 – NEurope; POR-009 – SWEurope; ALG-004 – Africa. Genes are color coded (Green – integrase; red – holin; blue – core (≥40% identity with presence in ≥ 90% of complete prophage genomes; integrase also within this group); gray – other genes). The predicted promoters are depicted with red arrows and coded with letters. The predicted gene function is shown (Grey letters for transcriptional regulators).

(b) Schematic of proposed lytic and Lysogenic cycles, based on representation of the ALG-004 prophage genome. 1: Choosing between lytic/lysogenic cycles upon infection. The repressor MarR regulates the events governing lysogeny versus lytic cycle, hypothetically binding to an AT-rich palindromic motif. The yellow hexagons contains the number of TTAA sites in the promoter region, where MarR can potentially bind, impeding gene transcription under the control of these promoters, leading to lysogeny. When MarR is not expressed in sufficient amounts, or is inactivated leads to the lytic pathway. MarR may also prevent infection by the same phage. 2: Switch to the lytic cycle. The putative histidine kinase (HK) hypothetically receives a stimulus (green arrow), inducing autophosphorylation of HK. The HK acts as the primary sensor, then the phosphate is transferred to other transcription factors to directly regulate the expression of a series of genes required for the lytic cycle. The putative phosphorylation of MarR (probable cys-phosphorylation of MarR encoded by gene 5, contains 8 cysteine residues susceptible to phosphorylation) may inactivate the repressor leading to the lytic cycle. 3: The full lytic cycle. The ArsR transcriptional regulator (homo-dimer) may regulate late gene expression. Phosphorylated ArsR may control transcription of genes beyond their baseline levels. The genes under the control of the transcriptional regulator NusG (including the lysis-cassette genes that lead to the bacterium lysis at the end of the lytic cycle) may have accelerated transcription elongation rate by suppression of specific transcription pause sites (green arrows).

Pseudogenes, often considered nonfunctional relics of once-active genes, can arise through various processes, such as gene duplication, genomic rearrangements, population bottlenecks, and genetic drift.^36^ These nonfunctional genes accumulate mutations over time that disrupt their ability to be transcribed or translated into functional proteins. The proportion of pseudogenes per 30kb genome segment was significantly higher among prophage genes, either complete (average 2.51; SD, 2.21; IQR, 2.86 pseudogenes/30kb) or incomplete (5.64; SD, 10.02; IQR, 8.64), than within bacterial genes (1.41; SD, 0.26; IQR, 0.31; Mann-Whitney U-test p-value <.0001, respectively). These pseudogenes carried internal stop codons, possessed frameshift mutations, and/or were incomplete. An increased presence of pseudogenes in the mobile genetic pool has been previously observed as well.^37^ Moreover, the proportion of pseudogenes is also significantly higher in incomplete than in complete prophages (Mann-Whitney U-test p-value <.0001). This observation suggests that *H. pylori* prophages suffer pseudogenization, which may hamper switching toward a lytic cycle and prophage arrest within the bacterial genome. This is compatible with the reductive evolution of the vertically inherited prophage regions, being even more evident in prophage remnants. An increased presence of pseudogenes has been found in intracellular bacteria that rely on exploiting host metabolites.^37,38^ A parallel with intracellular bacteria can be made for prophages that benefit from host replication for propagation. Pseudogenization may be a way to purge prophages from the bacterial genome, first these regions get retained (no longer able to follow a lytic cycle), but ultimately leading to gene deletion and genome reduction. The link between pseudogene prevalence and prophages could be affected by confounders like the dynamic nature of genomes and the potential of gene reactivation, underscoring the need to address these factors in future research.

### Prophage gene function provides clues to a model for lytic and lysogenic cycles

Based on the observed architectural genome synteny organization and the prediction of gene function of the 147 pangenome genes, we developed a model for the regulation of lytic and lysogenic cycles (Figure 4, Supplementary Figure S5, Table S6). Two prophage coding sequences toward the 5’ end of the prophage genome had homology with the MarR-like family of transcriptional regulators, placed downstream of the integrase gene, further pointing to a role in the lytic/lysogenic switch (genes #5 and #7, alias for genes numbered gff.5 and gff.7 in Supplementary Table S6). Members of this transcriptional regulator family are usually dimers that respond to chemical signals and stresses, converting them into changes in gene activity.^39^ We hypothesize that these MarR like transcriptional regulators may govern lysogenic and lytic cycles by binding to AT-rich palindromic motifs (which were found in prophage genomes near promoter regions), repressing transcription and controlling RNA polymerase expression of the genes under the control of these promoters (Figure 4), favoring the lysogenic cycle. In contrast, when MarR is not expressed in sufficient amounts, or is inactivated, it may lead to the lytic pathway. Importantly, the control of lysogeny by a MarR like repressor (localized upstream of the integrase gene) has been proposed for a T7-like prophage exhibiting a lysogenic cycle.^40^ Another key player in the induction of the lytic cycle appears to be a putative histidine kinase (HK)-like protein (Supplementary Table S6, gene #18). We propose that stimulation by HK may induce its autophosphorylation. The HK may act as the primary sensor, transferring the phosphate to other transcription factors to directly regulate the expression of a series of genes required for the lytic cycle. The putative phosphorylation of MarR-like transcriptional regulator (probable Cys-phosphorylation of MarR encoded by gene #5, which contains eight cysteine residues susceptible to phosphorylation or His-phosphorylation of the nine histidine residues; and gene #7, containing four cysteine residues) may inactivate the repressor leading to the lytic cycle. Usually, HK is involved in signal transduction across the cellular membrane by phosphotransfer and phosphatase activity. This HK-like enzyme may induce the host to phosphorylate other response regulator, changing gene expression. An HK-like gene associated with a quorum sensing system has been reported in a *Clostridium difficile* prophage.^41,42^ Thus, an HK-like gene may be involved in a quorum sensing mechanism just leading to phage release in the presence of neighboring cells in sufficient numbers to be infected. An argument favoring the association of phage induction and quorum sensing is that a high concentration of a susceptible host increases the advantages of phage lysis. High cell density detection by quorum sensing has been shown to play a role in prophage induction, either increasing,^43^ or decreasing^44^ it. A third ArsR-like transcriptional regulator, which is a response regulator (Supplementary Table S6, gene #28) may regulate late gene expression. The phosphorylated ArsR-like may control the transcription of genes beyond their baseline levels. A last regulator was identified, a NusG-like, similar to the N protein in phage λ (Supplementary Table S6, gene #34). The genes under the control of the transcriptional regulator NusG-like, including the lysis-cassette genes that lead to the bacterium lysis at the end of the lytic cycle, may have accelerated transcription elongation rate by suppressing specific transcription pause sites.

Other relevant genes unrelated to the switch of lytic/lysogenic cycle were also identified, remarkably with a gene organization based on function: lysogeny; replication; genome segregation; phage structure; phage assembly; genome packing; and cell lysis (Figure 4). In the lysogenic module, the integrase and MarR-like transcriptional regulators are found; the replication module presents a DNA helicase and a DNA primase; the segregation module presents a chromosome segregation protein with a DUF3519 domain identical to a bacterial gene (see above), which may be relevant for equipartition of episomal phages during cell division. Indeed, *H. pylori* has a homologous bacterial gene, and often incomplete prophages are fragmented at this gene, frequently occurring genome rearrangement at this gene level, pointing to homologous recombination between phage and bacterial genes. Two assembly modules follow, and noteworthy phages from SWEurope and Africa present more features (Supplementary Figure S5) with homology with structural phage proteins. These may explain the smaller phage tail length observed for phages like KHP30^24^ (EastAsia) than for phiHP33^11^ (Africa1). Finally, the lysis module presents the genes associated with cell burst and progeny release.

### H. pylori *prophages have a small but relevant cargo gene repertoire*

A prophage cargo gene is a gene within its genome that is not necessary for phage replication, assembly or virion structure. These genes, which can involve toxin production, antibiotic resistance, or metabolic pathways, may provide benefits to either the host bacterium or the phage itself. We could identify cargo genes within prophage sequences, even in complete prophages (Supplementary Table S7). These cargo genes are not essential for the function of the phage, but were present within the prophage element. The cargo genes include *tfs* genes; toxin-antitoxin systems; methyltransferase (an *N*-6 DNA methylase); or bacterial gene blocks. Of note, several prophage coding regions are identified as hypothetical proteins, and we must not dismiss that some of these may also constitute cargo genes that introduce novel properties to the phage and bacteria. The *tfs* plasticity zone clusters have been described as a virulence factor.^35^ Thus, the *tfs* cargo genes may contribute to the virulence of the strain. Toxin-antitoxin systems have only been briefly described in *H. pylori*,^45^ and are here described for the first time in the context of the prophage genome as the most common cargo gene. Toxin-antitoxin systems are prevalent in bacterial chromosomes and plasmids, serving various functions that span from stabilizing plasmids to facilitating biofilm formation and promoting bacterial persistence. In these systems, the toxin’s harmful effects are neutralized by the presence and activity of an antitoxin, which can be either an RNA or a protein.^46–48^ The presence of toxin-antitoxin may produce a post-segregational killing effect, favoring prophage maintenance. This phenomenon has been reported for *Escherichia coli* prophage P1.^49^ When these addiction genes are lost, the cell’s reservoir of antitoxin diminishes, enabling the toxin to exert its harmful effects on the cell.^50^ The bacterial gene blocks within a prophage genome sets the stage for phage transduction in *H. pylori*, in which bacteriophages transfer genetic material from one bacterium to another. This bacterial gene block appears to have resulted from a prophage genome inversion. Phage cargo genes can play a critical role in bacterial evolution, as they facilitate the exchange of genetic information between different bacterial strains, and may contribute to bacteria adapting to new environments and acquiring new traits.

## Conclusion

In this study, we have meticulously characterized the prophages of *H. pylori* within the high-quality *Hp*GP dataset. Prophage genes were more common than previously reported (29.5%) but decreased to about a third (9.5%) for complete prophages. *H. pylori* prophages present specific populations, thus shaping bacterial ecology. Prophage prevalence varies with geography and *H. pylori* ancestry, representing a complex interplay of ecological, evolutionary, and genetic factors. The *Hp*GP set revealed that prophage sequences are often fragmented and scattered across the *H. pylori* chromosome. This dispersion of prophage elements due to bacterial genome rearrangement disrupts the typical and likely essential modular organization required for the prophage to complete a lytic cycle. Likewise, merging with other mobile elements also disrupts sequences. These prophage genome rearrangements, merging with other mobile elements, and pseudogenization appear to be new players in the arms race between bacteria and phage. While functional annotation is challenging for phage genes, our analysis successfully determined the gene function for 61.2% of the prophage pangenome, which consists of 147 genes. Notably, this included key genes that enabled the formulation of an in silico-derived hypothesis regarding the switch model between lysis and lysogeny. This publicly available prophage catalog, together with its annotated pangenome, provide a necessary foundation for future experiments to untangle prophage evolution, ecology, function, regulation, phage–host interaction, and potential application in phage therapy.

## Material and methods

### Sample acquisition

Our analysis was based on 1011 *H. pylori* genomes from the *Hp*GP, which represent 50 countries. The sample acquisition details as well as bacterial isolation, DNA extraction, library preparation, SMRT/PacBio sequencing, data QC, and *de novo* assembly can be found in the study conducted concurrently with the present work.^51^ The *Hp*GP includes 12 countries from which no *H. pylori* genome sequences have previously been published. Collecting material from diverse geographical regions poses significant logistical challenges, and while sample groups could ideally be more diverse, the inclusion of 1011 genomes from the *Hp*GP represents the best possible representation given these constraints.

### Prophage identification

Prophages were identified in the genomes using the default options of BLASTn,^52^ and previous *H. pylori* identified prophages^25^ as a query. BLASTn between the prophage query and the bacterial chromosome identifies sequences with homology. However, due to sequence variability, this matching is not always correctly delimited in the bacterial chromosome. Thus, all hits were manually investigated so that the prophage region could be delimited, independently of query cover since we were also targeting incomplete prophages. The coding regions upstream and downstream of the identified regions were investigated using BLASTn against the nucleotide database until genes of bacterial origin were found, thus delimiting the prophage region. To perform the manual curation, the sequence flanking the prophage on each side is investigated by analyzing the following genes to verify whether they were bacterial or phage-related, using BLASTn against the nucleotide database of NCBI. If the analyzed genes are found to be phage-related, they are added to the prophage; otherwise, they help in delimiting the prophage within the bacterial genome. This was done until we found a bacterial gene that delimits the prophage sequence. To ensure that this bacterial gene was not a cargo gene among other bacterial genes, we verified the absence of another prophage in the immediate vicinity, as well as the gene order of the prophage, since prophage present genome synteny (by visualizing the annotated prophages in the Geneious 8 software). This analysis was performed for the two insertion sites of the prophage at the 5’ and 3’ ends. Additionally, using the annotated genome files a search for specific bacteriophage protein annotations were performed, using the keywords, “capsid,” “virion,” “lysin,” “holin,” “tail,” “structur*,” “integrase,” and “portal.” The hits were investigated using BLASTn and BLASTp and the prophage regions delimited. Furthermore, to verify for the presence of novel prophages without homology to the query used or the word search, we have used the phage detection algorithm PhiSpy,^19^ which is designed for *de novo* discovery of phage regions. *H. pylori* prophages described as complete typically have a size over 20 Kb, whereas remnant prophages have sizes much lower than this cutoff. Contiguous prophage regions longer than 20 kb with a prophage gene composition delimiting the sequence were marked as complete; while those with shorter sequences, or absence of phage genes delimiting the sequence, or situations where most genes were not phage-related were labeled incomplete. The locus tag of the bacterial genes at 5’ and 3’ ends delimiting the prophages were registered to evaluate integration site predominance. Python libraries numpy, matplotlib and scipy were used for box-plot, linear regression, chi-square or Fisher's exact test, and the Mann-Whitney U-test determination (genome size; GC content; number of pseudogenes identified during annotation). Certain assumptions of linear regression models, such as normality and homogeneity of residuals, as well as linearity for quantitative predictors, were evaluated using the Shapiro–Wilk test. We have done the Shapiro–Wilk test to evaluate the normality assumption, and Levene’s test to assess the equality of variances among the groups, before proceeding with either Fisher’s exact test or the independent t-student test.

### Prophage populations and phylogenetic analysis

Prophage sequences containing concatenated integrase and holin genes, as well as complete prophage genomes, were aligned utilizing MAFFT version 7^53^ with default settings. Maximum-likelihood phylogenetic trees were generated from nucleotide alignments through fasttreeMP 2.1.11.^54^ The Interactive Tree Of Life^55^ was employed for the visualization and annotation of the resultant trees. Furthermore, a phylogenetic network concerning complete prophages was constructed using SplitsTree 4.10 software.^56^ This software serves as a robust tool for depicting both conflicting and coherent information contained within a dataset.

We inferred the prophage population using the integrase and holin genes to run STRUCTURE,^6,57^ using the admixture model and 30,000 iterations, preceded by a burn-in phase of 30,000 iterations. Multiple runs were executed for values of 2 ≤ K ≤ 9, and comparisons were made based on the highest mean log-likelihood values obtained. Utilization of these two genes for population determination enabled the incorporation of both complete and incomplete prophages that possess them.

For the complete prophages the population structure was determined using chromosome painting and fineSTRUCTURE algorithms as previously described.^8^ Genome-wide SNPs, extracted with SNP-sites,^58^ were grouped into segments using a co-ancestry matrix, detailing the count of segments from each donor to each recipient. ChromoPainter (version 0.04) conducted the chromosome painting algorithm individually for each recipient. Subsequently, fineSTRUCTURE (version 0.02) underwent 100,000 iterations of burn-in and Markov Chain Monte Carlo to cluster individuals based on the co-ancestry matrix.^57^ Also, for complete prophage genomes, the genetic diversity assessment among the prophage populations determined employed the PopGenome package^59^ within R. This involved calculating F_ST_ (fixation index) to assess genetic structure and quantify inter-subpopulation genetic variation within the overall population, determining nucleotide diversity for gauging polymorphism in both groups, and using Tajima’s D statistics to identify deviations from neutrality. Furthermore, a PCA was conducted using the function glPca from the adegenet package^60^ in R.

### Prophage pangenome and functional annotation

The prophage pangenome was determined using Roary,^61^ as previously described,^8^ setting −i (the minimum percentage identity of the shorter length for BLASTp) to 70. Each protein sequence of the pangenome was investigated for its three-dimensional structure using Alphafold2^62^ with default parameters for monomer assembly, Phyre2^63^ and SwissModel.^64^ BLASTp and Pfam searches were conducted as well for the protein sequences. Globally, this analysis gave insight into protein function, which is of paramount importance concerning the high number of coding sequences identified without attributed function. Phage promoters were predicted using PHIRE.^65^ GO terms were determined to provide structured controlled ontologies describing fundamental characteristics of gene products.^66^ RM system annotation and epigenetic details can be found at REBASE.^28^ According to the Pacific biosciences white paper on base modifications, PacBio sequencing is very good at finding the chemical modifications at N6-adenine and N4-methylcytosine that occur on DNA molecules, but has great limitations decoding 5-methylcytosine.

### Genome synteny and rearrangement analysis

The genome synteny was visualized with EasyFig^67^ and clinker.^68^ Additionally, during manual delimitation of prophage sequences any observed genome rearrangement, like inversions, were registered. Dot-plots of particular genes or regions were constructed using Geneious version 8.

## Contributions

Manuscript conception and design: FFV, MCC and CSR. Data analysis: FFV, RJR, and IK. Interpretation of results: FFV, RJR, IK, MCC, CSR. Manuscript writing: FFV. Data coordinator: Difei Wang. Editing of the manuscript: *Hp*GP Research Network, RJR, IK, MCC, and CSR. Sample acquisition: *Hp*GP Research Network. Conception, design, and coordination of the *Hp*GP: MCC and CSR.

## Supplementary Material

TableS2.xlsx

TableS6.xlsx

Supplementary information Gut Microbes Final.docx

## Data Availability

All HpGP genomes are publicly available in the NCBI GenBank repository, BioProject accession PRJNA529500.

## Authors and affiliations

**Pathogen Genome Bioinformatics and Computational Biology, Research Institute for Medicines, Faculty of Pharmacy, Universidade de Lisboa, Lisboa, Portugal**

Filipa F. Vale

**Instituto de Biosistemas e Ciências Integrativas, Faculdade de Ciências, Universidade de Lisboa, Lisboa, Portugal**

Filipa F. Vale

**Cancer Genomics Research Laboratory, Frederick National Laboratory for Cancer Research, Frederick, MD, USA**

Difei Wang, Belynda Hicks, Bin Zhu, Meredith Yeager, Amy Hutchinson, Kedest Teshome, Kristie Jones & Wen Luo

**Division of Cancer Epidemiology and Genetics, National Cancer Institute, Rockville, MD, USA**

Difei Wang, Alisa M. Goldstein, Nan Hu, Philip R. Taylor, Minkyo Song, Andrés J. Gutiérrez-Escobar, Kai Yu, Bin Zhu, Christian C. Abnet, Stephen J. Chanock, M. Constanza Camargo & Charles S. Rabkin

**Department of Medicine, Vanderbilt University Medical Center, Nashville, TN, USA**

Judith Romero-Gallo, Uma Krishna, Richard M. Peek Jr, M. Blanca Piazuelo, Keith T. Wilson, John T. Loh & Timothy L. Cover

**Veterans Affairs Tennessee Valley Healthcare System, Nashville, TN, USA**

Timothy L. Cover, Keith T. Wilson

**Department of Natural and Life Sciences, Faculty of Sciences, University of Algiers 1 Benyoucef Benkhedda, Algiers, Algeria**

Naïma Raaf

**Department of Gastroenterology, Dhaka Medical College and Hospital, Dhaka, Bangladesh**

Hafeza Aftab

**Department of Environmental and Preventive Medicine, Oita University Faculty of Medicine, Yufu, Japan**

Junko Akada, Takashi Matsumoto & Yoshio Yamaoka

**Department of Pathobiology, Pharmacology and Zoological Medicine, Faculty of Veterinary Medicine, Ghent University, Ghent, Belgium**

Freddy Haesebrouck

**A.C.Camargo Cancer Center, São Paulo, São Paulo, Brazil**

Thais F. Bartelli, Diana Noronha Nunes, Adriane Pelosof, Claudia Zitron Sztokfisz & Emmanuel Dias-Neto

**Núcleo de Pesquisas em Oncologia, Universidade Federal do Pará, Belém, Pará, Brazil**

Paulo Pimentel Assumpção

**Medical University of Sofia, Sofia, Bulgaria**

Ivan Tishkov

**Faculty of Medicine and Dentistry, Department of Medicine, University of Alberta, Edmonton, AB, Canada**

Karen J. Goodman, Janis Geary, Taylor J. Cromarty & Nancy L. Price

**Department of Biomedical and Molecular Sciences, Queen’s University, Kingston, ON, Canada**

Douglas Quilty

**Department of Hematology and Oncology, Faculty of Medicine, Pontificia Universidad Católica de Chile, Santiago, Chile**

Alejandro H. Corvalan

**Department of Pediatric Gastroenterology and Nutrition, Faculty of Medicine, Pontificia Universidad Católica de Chile, Santiago, Chile**

Carolina A. Serrano

**Department of Gastroenterology, Faculty of Medicine, Pontificia Universidad Católica de Chile; and Center for Cancer Prevention and Control (CECAN), Santiago, Chile**

Robinson Gonzalez & Arnoldo Riquelme

**Facultad de Ciencias Biológicas, Universidad de Concepción, Concepción, Chile**

Apolinaria García-Cancino & Cristian Parra-Sepúlveda

**Hospital Hanga Roa, Easter Island, Chile**

Francisco Castillo

**Grupo de Investigación en Biología del Cáncer, Instituto Nacional de Cancerología, Bogotá DC, Colombia**

Maria Mercedes Bravo

**Departamento de Biología, Universidad de Nariño, Pasto, Nariño, Colombia**

Alvaro Pazos

**Escuela de Medicina, Universidad del Valle, Cali, Valle, Colombia**

Luis E. Bravo

**Division of Comparative Medicine, Department of Biological Engineering, Massachusetts Institute of Technology, Cambridge, MA, USA**

James G. Fox

**Instituto de Investigaciones en Salud, Universidad de Costa Rica, San Jose, Costa Rica**

Vanessa Ramírez-Mayorga & Silvia Molina-Castro

**Laboratorio de Patología General y Gastrointestinal (LABGIPAT), San Jose, Costa Rica**

Sundry Durán-Bermúdez

**Servicio de Gastroenterología y Endoscopía Digestiva, Hospital Clínica Bíblica, San Jose, Costa Rica**

Christian Campos-Núñez & Manuel Chaves-Cervantes

**Faculty of Medicine, Osaka Metropolitan University, Osaka, Japan**

Evariste Tshibangu-Kabamba

**Faculty of Medicine, University of Mbuji-Mayi, Mbuji Mayi, Kasai-Oriental, Democratic Republic of the Congo**

Evariste Tshibangu-Kabamba & Ghislain Disashi Tumba

**Faculty of Medicine, University of Kinshasa, Kinshasa, Democratic Republic of the Congo**

Antoine Tshimpi-Wola, Patrick de Jesus Ngoma-Kisoko & Dieudonné Mumba Ngoyi

**Department of Parasitology, National Institute of Biomedical Research, Kinshasa, Democratic Republic of the Congo**

Dieudonné Mumba Ngoyi

**Instituto de Microbiología y Parasitología, IMPA, Universidad Autónoma de Santo Domingo, UASD, Santo Domingo, Dominican Republic**

Modesto Cruz & Celso Hosking

**Dominican-Japanese Digestive Disease Center, Dr Luis E. Aybar Health and Hygiene City, Santo Domingo, Dominican Republic**

José Jiménez Abreu

**Bordeaux Institute of Oncology, BRIC U1312, INSERM, Bordeaux, and National Reference Center for Campylobacters & Helicobacters, CHU de Bordeaux, Bordeaux, France**

Christine Varon, Lucie Benejat, Quentin Jehanne, Philippe Lehours & Francis Megraud

**Medical Research Council Unit, The Gambia at the London School of Hygiene & Tropical Medicine, Banjul, The Gambia**

Ousman Secka

**Department of Gastroenterology, Hepatology and Infectious Diseases, Otto-von-Guericke University Magdeburg, Magdeburg, Germany**

Alexander Link & Peter Malfertheiner

**Department of Biochemistry, School of Biological Sciences, University of Cape Coast, Cape Coast, Central Region, Ghana**

Michael Buenor Adinortey

**Department of Internal Medicine and Therapeutics, School of Medical Sciences, University of Cape Coast, Cape Coast, Central Region, Ghana**

Ansumana Sandy Bockarie

**Department of Molecular Biology and Biotechnology, School of Biological Sciences, University of Cape Coast, Cape Coast, Central Region, Ghana**

Cynthia Ayefoumi Adinortey

**Department of Biology Education, Faculty of Science Education, University of Education, Winneba, Ghana**

Eric Gyamerah Ofori

**Laboratory of Medical Microbiology, Hellenic Pasteur Institute, Athens, Greece**

Dionyssios N. Sgouras & Beatriz Martinez-Gonzalez

**Department of Gastroenterology, Alexandra Hospital, Athens, Greece**

Spyridon Michopoulos

**Department of Gastroenterology, Athens Medical, P. Faliron Hospital, Athens, Greece**

Sotirios Georgopoulos

**Facultad de Ciencias Médicas, University of San Carlos of Guatemala, Guatemala City, Guatemala**

Elisa Hernandez

**Departamento de Medicina Interna, Hospital de Occidente, Santa Rosa de Copán, Honduras**

Ricardo L. Dominguez

**School of Medicine, University of Alabama at Birmingham (UAB), Birmingham, AL, USA**

Douglas R. Morgan

**Landspítali – The National University Hospital of Iceland, Reykjavík, Iceland**

Hjördís Harðardóttir, Anna Ingibjörg Gunnarsdóttir, Hallgrímur Guðjónsson, Jón Gunnlaugur Jónasson & Einar S. Björnsson

**University of Iceland, Reykjavík, Iceland**

Jón Gunnlaugur Jónasson & Einar S. Björnsson

**Department of Microbiology, Kasturba Medical College, Manipal Academy of Higher Education, Manipal, Karnataka, India**

Mamatha Ballal & Vignesh Shetty

**Department of Medicine, University of Cambridge, Cambridge, UK**

Vignesh Shetty

**Universitas Airlangga, Surabaya, East Java, Indonesia**

Muhammad Miftahussurur, Titong Sugihartono, Ricky Indra Alfaray, Langgeng Agung Waskito & Kartika Afrida Fauzia

**University of Indonesia, Jakarta, Indonesia**

Ari Fahrial Syam & Hasan Maulahela

**Digestive Disease Research Institute, Tehran University of Medical Sciences, Tehran, Iran**

Reza Malekzadeh & Masoud Sotoudeh

**Clinical Microbiology Laboratory and Research Institute, Tzafon Medical Center, affiliated with Azrieli Faculty of Medicine, Bar Ilan University, Poriya, Israel**

Avi Peretz & Maya Azrad

**Azrieli Faculty of Medicine, Bar Ilan University, Safed, Israel**

Avi Peretz & Avi On

**Pediatric Gastroenterology and Nutrition Unit, Tzafon Medical Center, Poriya, Israel**

Avi On

**Unit of Immunopathology and Oncological Biomarkers, Centro di Riferimento Oncologico di Aviano, Aviano, Italy**

Valli De Re & Stefania Zanussi

**Unit of Oncological Gastroenterology, Centro di Riferimento Oncologico di Aviano (CRO) IRCCS, Italy; Department of Medical, Surgical and Health Sciences, University of Trieste, Italy**

Renato Cannizzaro

**Unit of Pathology, Centro di Riferimento Oncologico di Aviano (CRO) IRCCS, Italy;**

**Department of Medical, Surgical and Health Sciences, University of Trieste, Italy**

Vincenzo Canzonieri

**Department of Gastroenterology and Metabolism, Nagoya City University Graduate School of Medical Sciences, Nagoya, Japan**

Takaya Shimura

**School of Medicine, Kyorin University, Mitaka, Tokyo, Japan**

Kengo Tokunaga, Takako Osaki & Shigeru Kamiya

**Faculty of Medicine, Jordan University of Science and Technology, Ar-Ramtha, Jordan**

Khaled Jadallah & Ismail Matalka

**Research Institute of Life and Health Sciences, Higher School of Medicine, Kokshetau University named after Sh. Ualikhanov, Kokshetau, Kazakhstan**

Igissin Nurbek Sagynbekuly

**Central Asian Institute for Medical Research, Astana, Kazakhstan**

Igissin Nurbek Sagynbekuly

**Asian Pacific Organization for Cancer Prevention, Bishkek, Kyrgyzstan**

Igissin Nurbek Sagynbekuly

**Kyrgyz State Medical Academy, Bishkek, Kyrgyzstan**

Mariia Satarovna Moldobaeva & Attokurova Rakhat

**Center for Gastric Cancer, National Cancer Center, Goyang, South Korea**

Il Ju Choi

**Department of Internal Medicine, Chung-Ang University Hospital, Seoul, South Korea**

Jae Gyu Kim

**College of Medicine, Seoul National University, Seoul, South Korea**

Nayoung Kim

**Institute of Clinical and Preventive Medicine, University of Latvia, Riga, Latvia**

Mārcis Leja, Reinis Vangravs, Ģirts Šķenders, Aiga Rūdule & Ilze Kikuste

**Digestive Diseases Centre GASTRO, Riga, Latvia**

Mārcis Leja & Aigars Vanags

**Riga East University Hospital, Riga, Latvia**

Mārcis Leja, Ģirts Šķenders & Dace Rudzīte

**Department of Gastroenterology, Institute for Digestive Research, Medical Academy, Lithuanian University of Health Sciences, Kaunas, Lithuania**

Juozas Kupcinskas, Jurgita Skieceviciene, Laimas Jonaitis, Gediminas Kiudelis, Paulius Jonaitis, Vytautas Kiudelis & Greta Varkalaite

**Department of Medical Microbiology, Faculty of Medicine, Universiti Malaya, Kuala Lumpur, Malaysia**

Jamuna Vadivelu, Mun Fai Loke & Kumutha Malar Vellasamy

**Medical Education Research and Development Unit, Faculty of Medicine, Universiti Malaya, Kuala Lumpur, Malaysia**

Jamuna Vadivelu

**Departamento de Patología, Instituto Nacional de Cancerología, Mexico City, Mexico**

Roberto Herrera-Goepfert

**Servicio de Endoscopía, Instituto Nacional de Cancerología, Mexico City, Mexico**

Juan Octavio Alonso-Larraga

**Defence Services General Hospital, Yangon, Yangon, Yangon Region, Myanmar**

Than Than Yee & Kyaw Htet

**Nippon Medical School, Tokyo, Japan**

Takeshi Matsuhisa

**Department of Gastroenterology, Maharajgunj Medical Campus, Tribhuvan University Teaching Hospital, Kathmandu, Nepal**

Pradeep Krishna Shrestha

**Division of Health Sciences, Abu Dhabi Women’s Campus, Higher Colleges of Technology, Abu Dhabi, United Arab Emirates**

Shamshul Ansari

**Department of Community Medicine, Babcock University, Ilishan, Ogun State, Nigeria**

Olumide Abiodun

**Department of Medicine, Babcock University, Ilishan, Ogun State, Nigeria**

Christopher Jemilohun

**Department of Medicine, College of Medicine, University of Ibadan, Ibadan, Oyo, Nigeria**

Kolawole Oluseyi Akande

**Department of Nursing, Crescent University, Abeokuta, Ogun State, Nigeria**

Oluwatosin Olu-Abiodun

**University Teaching Hospital, University of Jos, Jos, Plateau, Nigeria**

Francis Ajang Magaji

**University of Calabar Teaching Hospital, Calabar, Cross River, Nigeria**

Ayodele Omotoso & Uchenna Okonkwo

**Department of Surgery, University of Nigeria Teaching Hospital, Ituku-Ozalla, Enugu State, Nigeria**

Chukwuemeka Chukwunwendu Osuagwu

**Department of Internal Medicine, Federal Medical Center Abeokuta, Abeokuta, Ogun State, Nigeria**

Opeyemi O. Owoseni

**Faculty of Health Sciences, Universidad Científica del Sur, Lima, Peru**

Carlos Castaneda

**Departamento de investigación, Instituto Nacional de Enfermedades Neoplasicas, Lima, Peru**

Miluska Castillo

**Universidad Peruana Cayetano Heredia, Lima, Peru**

Billie Velapatino

**Bloomberg School of Public Health, Johns Hopkins University, Baltimore, MD, USA**

Robert H. Gilman

**Department of Microbiology, Wroclaw Medical University, Wrocław, Poland**

Paweł Krzyżek & Grażyna Gościniak

**Department of Surgery Teaching, Wroclaw Medical University, Wrocław, Poland**

Dorota Pawełka

**Department of Pharmaceutical Microbiology, Medical University of Lublin, Lublin, Poland**

Izabela Korona-Glowniak

**Department of Gastroenterology with Endoscopic Unit, Medical University of Lublin, Lublin, Poland**

Halina Cichoz-Lach

**Instituto Nacional de Saúde Dr. Ricardo Jorge, Lisboa, Portugal**

Monica Oleastro

**Instituto de Patologia e Imunologia Molecular da Universidade do Porto, Porto, Portugal**

Ceu Figueiredo, Jose C. Machado & Rui M. Ferreira

**Instituto de Investigação e Inovação em Saúde, Universidade do Porto, Porto, Portugal**

Ceu Figueiredo, Jose C. Machado & Rui M. Ferreira

**Faculdade de Medicina da Universidade do Porto, Porto, Portugal**

Ceu Figueiredo & Jose C. Machado

**Department of Pancreatic, Biliary and Upper Digestive Tract Disorders, A. S. Loginov Moscow Clinical Scientific Center, Moscow, Russia**

Dmitry S. Bordin

**Department of General Medical Practice and Family Medicine, Tver State Medical University, Moscow, Russia**

Dmitry S. Bordin

**Department of Propaedeutic of Internal diseases and Gastroenterology, Russian University of Medicine, Moscow, Russia**

Dmitry S. Bordin

**Department of Faculty Therapy and Gastroenterology, Omsk State Medical University, Omsk, Russia**

Maria A. Livzan

**Scientific Research Institute of Medical Problems of the North, Federal Research Centre “Krasnoyarsk Science Centre” of the Siberian Branch of Russian Academy of Science, Krasnoyarsk, Russia**

Vladislav V. Tsukanov

**Cancer Science Institute of Singapore, National University of Singapore, Singapore, Singapore**

Patrick Tan

**Cancer and Stem Cell Biology Program, Duke NUS Medical School, Singapore, Singapore**

Patrick Tan

**Genome Institute of Singapore, Agency for Science, Technology and Research, Singapore, Singapore**

Patrick Tan

**Department of Medicine, Yong Loo Lin School of Medicine, National University of Singapore, Singapore, Singapore**

Khay Guan Yeoh & Feng Zhu

**Department of Gastroenterology and Hepatology, National University Health System, Singapore, Singapore**

Khay Guan Yeoh

**Chris Hani Baragwanath Academic Hospital, Johannesburg, South Africa**

Reid Ally

**University of the Witwatersrand, Johannesburg, South Africa**

Reid Ally

**Max von Pettenkofer Institute of Hygiene and Medical Microbiology, Faculty of Medicine, LMU Munich, Munich, Germany**

Rainer Haas & Wolfgang Fischer

**Department of Microbiology, Hospital Universitario Donostia-Instituto de Investigacion Sanitaria Biogipuzkoa, San Sebastian, Spain**

Milagrosa Montes, María Fernández-Reyes, Esther Tamayo & Jacobo Lizasoain

**Department of Gastroenterology, Bioodonostia Health Research Institute – Donostia University Hospital, Universidad del País Vasco (UPV/EHU), Centro de Investigación Biomédica en Red de Enfermedades Hepáticas y Digestivas (CIBERehd), San Sebastian, Spain**

Luis Bujanda

**Digestive Diseases Unit, Parc Taulí Hospital Universitari. Institut d’Investigació i Innovació Parc Taulí (I3PT-CERCA), Universitat Autònoma de Barcelona, Barcelona, Spain**

Sergio Lario, María José Ramírez-Lázaro, Xavier Calvet & Eduard Brunet-Mas

**Centro de Investigación Biomédica en Red de Enfermedades Hepáticas y Digestivas, Instituto de Salud Carlos III, Madrid, Spain**

Sergio Lario, María José Ramírez-Lázaro, Xavier Calvet & Eduard Brunet-Mas

**Lozano Blesa University Clinic Hospital, Zaragoza, Spain**

María José Domper-Arnal & Sandra García-Mateo

**Aragon Health Research Institute, Zaragoza, Spain**

María José Domper-Arnal & Sandra García-Mateo

**Miguel Servet University Hospital, Zaragoza, Spain**

Daniel Abad-Baroja

**Hospital General de Granollers, Barcelona, Spain**

Pedro Delgado-Guillena

**Gastroenterology Department, Hospital Clínic of Barcelona, Centro de Investigación Biomédica en Red de Enfermedades Hepáticas y Digestivas, Facultat de Medicina i Ciències de la Salut, Universitat de Barcelona, Barcelona, Spain**

Leticia Moreira

**Hospital Universitari de Bellvitge, L’Hospitalet de Llobregat, Barcelona, Spain**

Josep Botargues

**Department of Gastroenterology, Hospital Universitario Central de Asturias, Oviedo, Asturias, Spain**

Isabel Pérez-Martínez & Eva Barreiro-Alonso

**Diet, Microbiota and Health Group, Instituto de Investigación Sanitaria del Principado de Asturias, Oviedo, Asturias, Spain**

Isabel Pérez-Martínez

**Farmacology Group, Instituto de Investigación Sanitaria del Principado de Asturias, Oviedo, Asturias, Spain**

Eva Barreiro-Alonso

**Instituto Universitario de Oncología del Principado de Asturias, Oviedo, Asturias, Spain**

Eva Barreiro-Alonso

**Hospital General Universitario Gregorio Marañón, Madrid, Spain**

Virginia Flores

**Gastroenterology Unit, Hospital Universitario de La Princesa, Instituto de Investigación Sanitaria Princesa, Madrid, Spain**

Javier P. Gisbert

**Universidad Autónoma de Madrid, Centro de Investigación Biomédica en Red de Enfermedades Hepáticas y Digestivas, Madrid, Spain**

Javier P. Gisbert

**Gastroenterology Department, Hospital Universitario de Navarra, Pamplona, Navarra, Spain**

Edurne Amorena Muro

**Hospital de Leon, Leon, Spain**

Pedro Linares & Laura Alcoba

**Institut of Biomedicine, University of León, Consortium for Biomedical Research in Epidemiology and Public Health, Leon, Spain**

Vicente Martin

**Instituto de Investigación Sanitaria INCLIVA, Hospital Clínico Universitario de Valencia, Valencia, Spain**

Tania Fleitas-Kanonnikoff

**Biochemistry Department, Faculty of Science, King Abdulaziz University, Jeddah, Saudi Arabia**

Hisham N. Altayeb

**Faculty of Medical laboratory Science, Sudan University of Science and Technology, Khartoum, Sudan**

Hisham N. Altayeb

**Centre for Translational Microbiome Research, Department of Microbiology, Tumor and Cell Biology, Karolinska Institutet, Solna, Sweden**

Lars Engstrand

**University of Skövde, Skövde, Sweden**

Helena Enroth

**Institute for Infectious Diseases, University of Bern, Bern, Switzerland**

Peter M. Keller

**Clinical Bacteriology/Mycology Unit, University Hospital Basel, Basel, Switzerland**

Peter M. Keller

**Institute of Medical Microbiology, University of Zurich, Zürich, Switzerland**

Karoline Wagner

**Clinic for Gastroenterology and Hepatology, University Hospital Zurich, Zürich, Switzerland**

Daniel Pohl

**College of Medicine, National Taiwan University, Taipei City, Taiwan**

Yi-Chia Lee, Jyh-Ming Liou & Ming-Shiang Wu

**Medical Microbiology Department, Cerrahpasa Medical Faculty, Istanbul University-Cerrahpasa, İstanbul, Türkiye**

Bekir Kocazeybek & Suat Sarıbas

**General Surgery Department, Cerrahpasa Medical Faculty, Istanbul University-Cerrahpasa, İstanbul, Türkiye**

İhsan Tasçı & Süleyman Demiryas

**Medical Pathology Department, Cerrahpasa Medical Faculty, Istanbul University-Cerrahpasa, İstanbul, Türkiye**

Nuray Kepil

**Staten Island University Hospital -Northwell Health, Staten Island, NY, USA**

Luis Quiel

**Carson Tahoe Regional Medical Center, Carson City, NV, USA**

Miguel Villagra

**White River Medical Center, Batesville, AR, USA**

Morgan Norton & Deborah Johnson

**Department of Medicine, Stanford University, Stanford, CA, USA**

Robert J. Huang & Joo Ha Hwang

**Albert Einstein College of Medicine, Bronx, NY, USA**

Wendy Szymczak, Saranathan Rajagopalan, Emmanuel Asare, William R. Jacobs Jr. & Haejin In

**Rutgers Cancer Institute, New Brunswick, NJ, USA**

Haejin In

**Georgia Cancer Center’s Biorepository, Augusta University, Augusta, Georgia**

Roni Bollag & Aileen Lopez

**Augusta University Medical Center, Augusta, Georgia**

Edward J. Kruse & Joseph White

**Section of Gastroenterology and Hepatology, Department of Medicine, Baylor College of Medicine, Houston, TX, USA**

David Y. Graham & Yoshio Yamaoka

**Enteric Diseases Laboratory Branch, Division of Foodborne, Waterborne, and Environmental Diseases, Centers for Disease Control and Prevention, Atlanta, GA, USA**

Charlotte Lane & Yang Gao

**Gi Care for Kids, LLC, Children’s Center for Digestive Healthcare, LLC, Atlanta, GA, USA**

Benjamin D. Gold

**University of Puerto Rico Comprehensive Cancer Center, San Juan, Puerto Rico**

Marcia Cruz-Correa & María González-Pons

**University of Puerto Rico Medical Sciences Campus, San Juan, Puerto Rico**

Marcia Cruz-Correa

**Gastrointestinal and Other Cancers Research Group, Division of Cancer Prevention, National Cancer Institute, Rockville, MD, USA**

Luz M. Rodriguez

**Department of Endoscopy, Cho Ray Hospital, Ho Chi Minh City, Vietnam**

Vo Phuoc Tuan, Ho Dang Quy Dung & Tran Thanh Binh

**Department of Hepatogastroenterology, 108 Military Central Hospital, Hanoi, Vietnam**

Tran Thi Huyen Trang & Vu Van Khien

**Sequencing Facility Bioinformatics Group, Bioinformatics and Computational Science Directorate, Frederick National Laboratory for Cancer Research, Frederick, MD, USA**

Xiongfong Chen & Yongmei Zhao

**Cancer Research Technology Program, Frederick National Laboratory for Cancer Research, Frederick, MD, USA**

Castle Raley, Bailey Kessing & Bao Tran

**Center for the Evolutionary Origins of Human Behavior, Kyoto University, Inuyama, Japan**

Yukako Katsura

**Instituto de Ciencias Biomédicas (ICBM), Facultad de Medicina, Universidad de Chile, Santiago, Chile**

Patricio Gonzalez-Hormazabal

**School of Life Sciences, University of Warwick, Coventry, UK**

Xavier Didelot

**Department of Biology & Biochemistry, University of Bath, Bath, UK**

Sam Sheppard

**Departamento de Genética, Ecologia e Evolução, Universidade Federal de Minas Gerais, Belo Horizonte, Brazil**

Eduardo Tarazona-Santos & Roxana Zamudio

**National Institute on Minority Health and Health Disparities, Bethesda, MD, USA**

Leonardo Mariño-Ramírez

**Division of Microbiology, Department of Biology, Friedrich-Alexander-Universität Erlangen-Nürnberg, Erlangen, Germany**

Steffen Backert

**Institute of Experimental Internal Medicine, Otto-von-Guericke-Universität Magdeburg, Magdeburg, Germany**

Michael Naumann

**Laboratory of Experimental Medicine and Pediatrics, Faculty of Medicine and Health Sciences, University of Antwerp, Antwerp, Belgium**

Annemieke Smet

**Department of Molecular Microbiology, Washington University School of Medicine, St. Louis, MO, USA**

Douglas E. Berg

**Genomics and Health Area, FISABIO – Public Health, Valencia, Spain**

Álvaro Chiner-Oms

**CIBER in Epidemiology and Public Health, Madrid, Spain**

Álvaro Chiner-Oms & Iñaki Comas

**Tuberculosis Genomics Unit, Instituto de Biomedicina de Valencia, Consejo Superior de Investigaciones Científicas, Valencia, Spain**

Iñaki Comas & Francisco José Martínez-Martínez

**Quadram Institute Bioscience, Norwich, UK**

Roxana Zamudio

**National Institute of Infectious Diseases, Tokyo, Japan**

Koji Yahara

**Center for Advanced Biotechnology and Medicine, Rutgers University, New Brunswick, NJ, USA**

Martin J. Blaser

**New England Biolabs, Ipswich, MA, USA**

Tamas Vincze, Richard D. Morgan & Richard J. Roberts

**Bacterial Pathogenesis and Antimicrobial Resistance Unit, National Institute of Allergy and Infectious Diseases, Bethesda, MD, USA**

John P. Dekker

**Unidad de Investigación en Enfermedades Infecciosas y Parasitarias, UMAE Pediatría, Instituto de Seguro Social, Mexico City, Mexico**

Javier Torres

**Department of Genetics, SOKENDAI University, Mishima, Shizuoka, Japan**

Mehwish Noureen

**National Center for Biotechnology Information, National Library of Medicine, National Institutes of Health, Bethesda, MD, USA**

Joshua L. Cherry

**Division of International Epidemiology and Population Studies, Fogarty International Center, National Institutes of Health, Bethesda, MD, USA**

Joshua L. Cherry

**Faculty of Information Science and Technology, Hokkaido University, Sapporo, Japan**

Naoki Osada

**Department of Molecular Oncology, Chiba University, Chiba, Japan**

Masaki Fukuyo

**Bioinformation and DDBJ Center, National Institute of Genetics, Mishima, Shizuoka, Japan**

Masanori Arita

**Instituto Nacional de Medicina Genómica, Ciudad de México, México**

Santiago Sandoval-Motta

**Consejo Nacional de Ciencia y Tecnologia, Cátedras CONACYT, Ciudad de México, México**

Santiago Sandoval-Motta

**Centro de Ciencias de la Complejidad, Universidad Nacional Autónoma de México, Ciudad de México, México**

Santiago Sandoval-Motta

**Department of Life Sciences and Biotechnology, University of Ferrara, Ferrara, Italy**

Rajiv Boscolo Agostini & Silvia Ghirotto

**Facultad de Ciencias Químicas, Universidad Autónoma de Chihuahua, Chihuahua, Chihuahua, México**

Zilia Y. Muñoz-Ramírez

**Centre for Microbes Development and Health, Institute Pasteur Shanghai, Shanghai, China**

Roberto C. Torres & Daniel Falush

**Department of Chemistry and Molecular Biology, University of Gothenburg, Gothenburg, Sweden**

Kaisa Thorell

**National Institute for Basic Biology, National Institutes of Natural Sciences, Aichi, Japan**

Ikuo Uchiyama

**Research Center for Micro-Nano Technology, Hosei University, Tokyo, Japan**

Ichizo Kobayashi
