# Supplementary material for: Gene content, phage cycle regulation model and prophage inactivation disclosed by prophage genomics in the Helicobacter pylori Genome Project
Source: Gut Microbes. 2024 Aug 12;16(1):2379440. doi: 10.1080/19490976.2024.2379440 (PMC11321410; doi:10.1080/19490976.2024.2379440)
Supplement: Supplementary information Gut Microbes Final.docx [file KGMI_A_2379440_SM2716.docx]

**Supplemental material**

Appendix to “**Gene content, phage cycle regulation model and prophage inactivation disclosed by prophage genomics in the *Helicobacter pylori* Genome Project**”

Filipa F. Vale, PhD, *Hp*GP Research Network, Richard J. Roberts, PhD, Ichizo Kobayashi, PhD, M. Constanza Camargo*, PhD, Charles S. Rabkin*, MD

* These authors jointly supervised this work.

**Table of contents**

[**Supplementary Figures** 1](#_Toc161747256)

[**Supplementary Tables** 5](#_Toc161747257)

# **Supplementary Figures**

**A.**


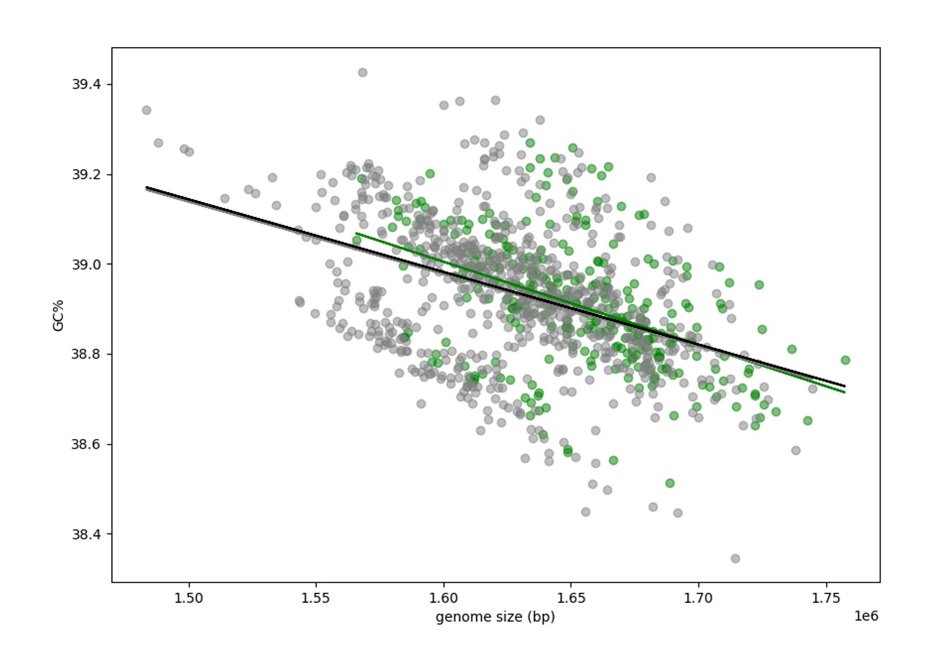


**B.**


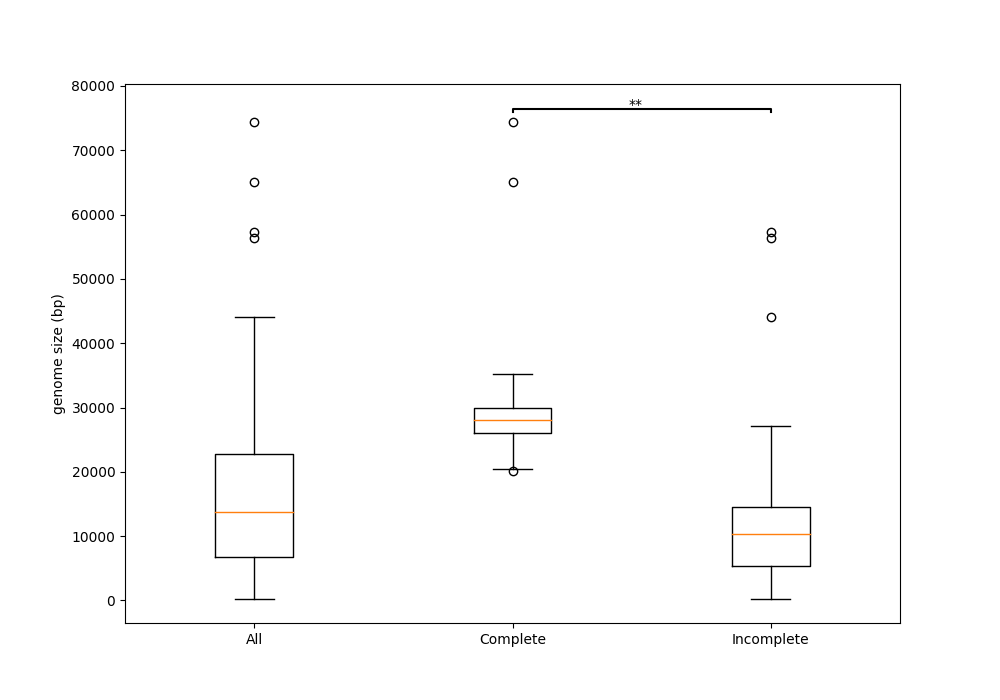


**C.**


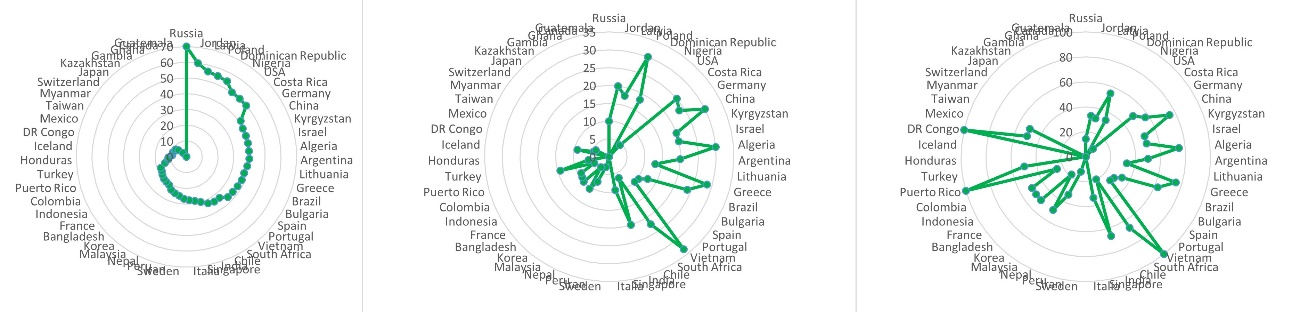


**Figure S1**. **A**. Genome size and GC content of *H. pylori* with and without prophages. Genomes with prophages are significantly larger (Mann-Whitney U test p <0.0001) with a tendency to smaller GC% content (t-student test p = 0.0153). Green: bacterial genome with prophage. Grey: bacterial without prophage. Reported values include beta coefficient estimate (-1.845e-06, 95% CI [-2.217e-06, -1.479e-06], p < 0.0001) and correlation coefficient (-0.464, 95% CI [-0.548, -0.369], p < 0.0001).**B**. Prophage genome size according to prophage completeness. Complete prophage (96 complete prophage sequences) are significantly larger (Mann-Whitney U test ** p <0.0001) than incomplete prophage sequences (272 complete incomplete prophage sequences). Outliers are defined as data points beyond 1.5 times the interquartile range from the first and third quartiles. **C**. Uneven prophage distribution by country (Fisher exact test p < 0.0001). From left to right: prevalence (percent) of genomes with prophage elements (complete and/or incomplete); complete prophage; and percent of the prophage elements that are complete.


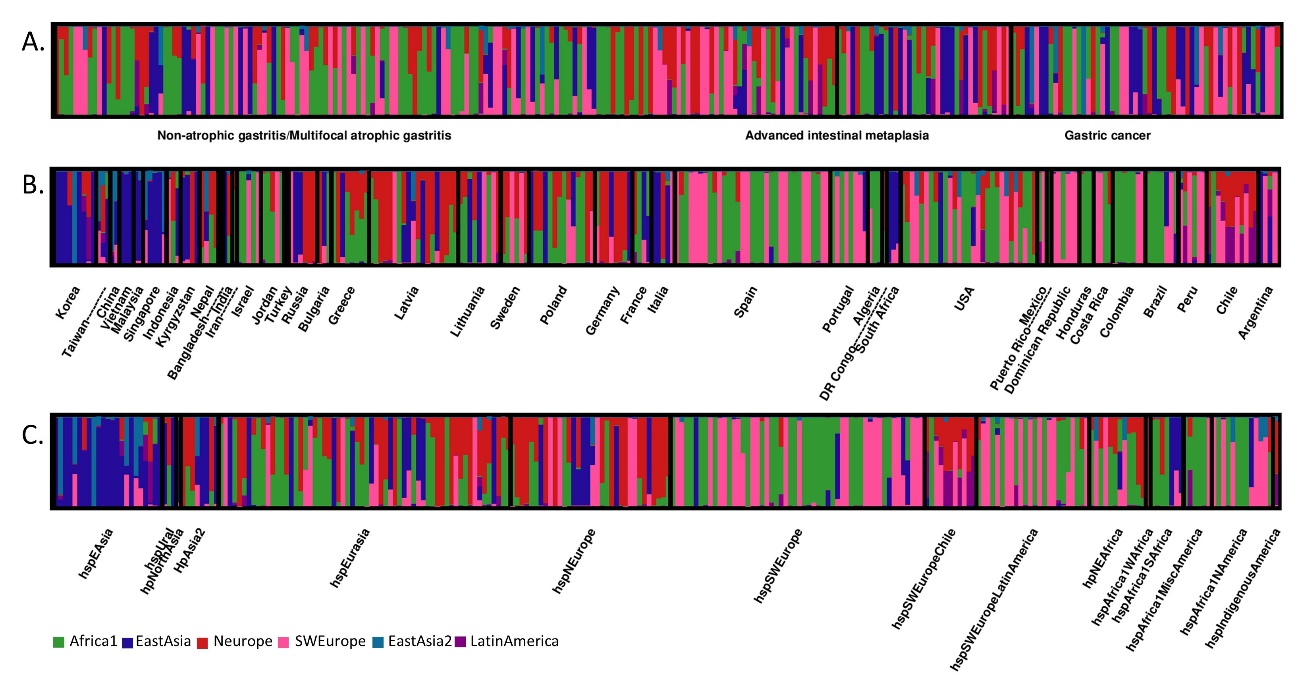


**Figure S2**. Agreement of prophage population with *H. pylori* ancestry and country of isolation, but not disease associated status. DISTRUCT plot of Bayesian population assigned using STRUCTURE and an admixture model for 6 populations considering 260 genomes containing the prophage integrase and holin genes. Each prophage is represented by a vertical line divided into K colored segments representing the membership coefficients in each cluster organized by **A**. disease associated with *H. pylori* isolate; **B**. country of origin and **C**. *H. pylori* fineSTRUCTURE ancestry.


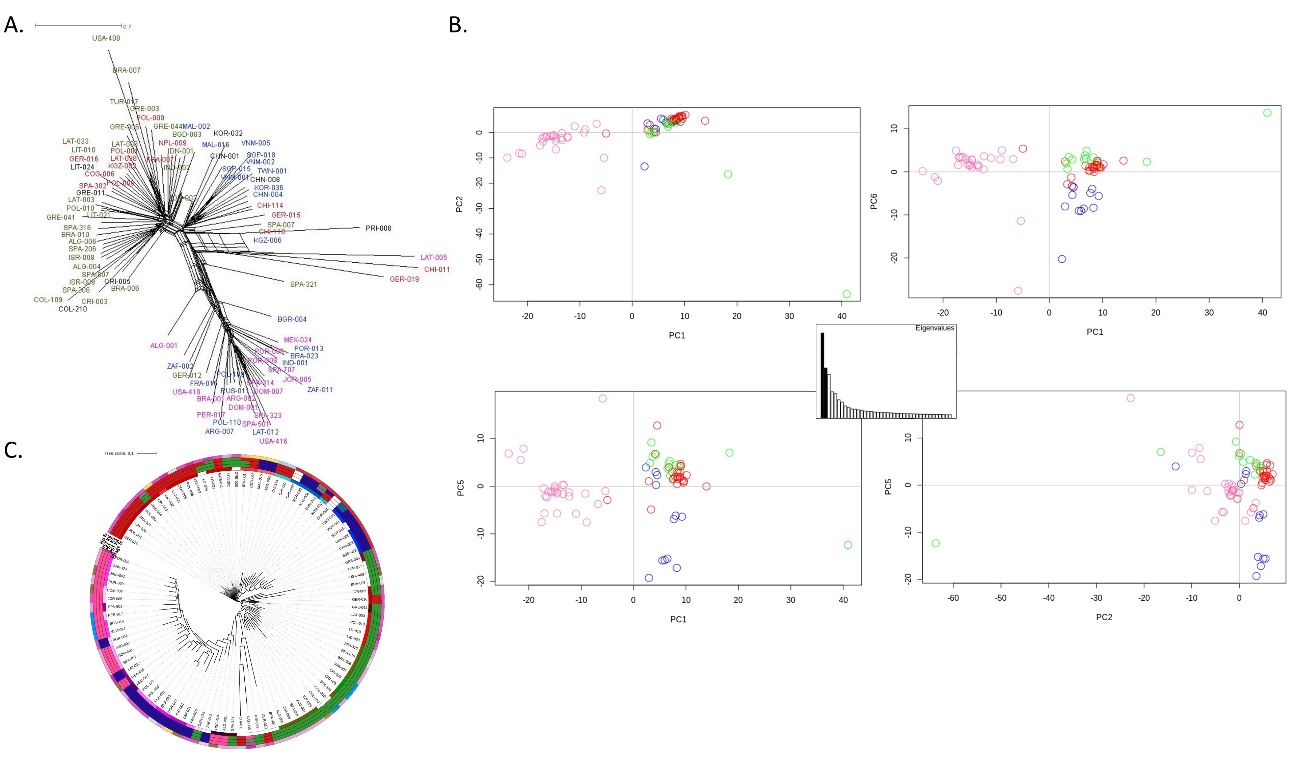


**Figure S3**. **A**. SplitsTree phylogenetic network reconstructed using the complete genome alignment of 95 complete prophages (complete prophages GRE-041 and GRE-046 are 100% identical, only one was kept). Tip colors correspond to those of Figure 2A. **B**. PCA analysis of 80 complete prophage genomes. PC1 vs PC2 separates SWEurope; PC1 vs PC6 separates EastAsia; PC1 vs PC5 separates Africa; and PC2 vs PC5 separates NEurope. Each circle represent a prophage genome colored as in Figure 1A for PST K=4. **C**. Maximum-likelihood phylogenetic trees from 95 complete prophage genome alignment. Circles from in to out: phageFS – population determined by fineSTRUCTURE coded as in Figure 2A; PST K4, PST K5 and PST K6 – populations determined by phage sequencing typing (integrase and holin) coded as in Figure 1A.

**Figure S4**. Global prophage genome alignments. A. Gene cluster alignment of complete prophages. Large figure available at:

<https://drive.google.com/file/d/1OfF50fdDjR2RpvpMxQG_RVGGZmFc5UKM/view?usp=share_link>

B. Gene cluster alignment of complete prophages. Large figure available at:

<https://drive.google.com/file/d/1Qyp0_bo-kdFi6SdkZHTAuYjJ7qDXsMw-/view?usp=share_link>


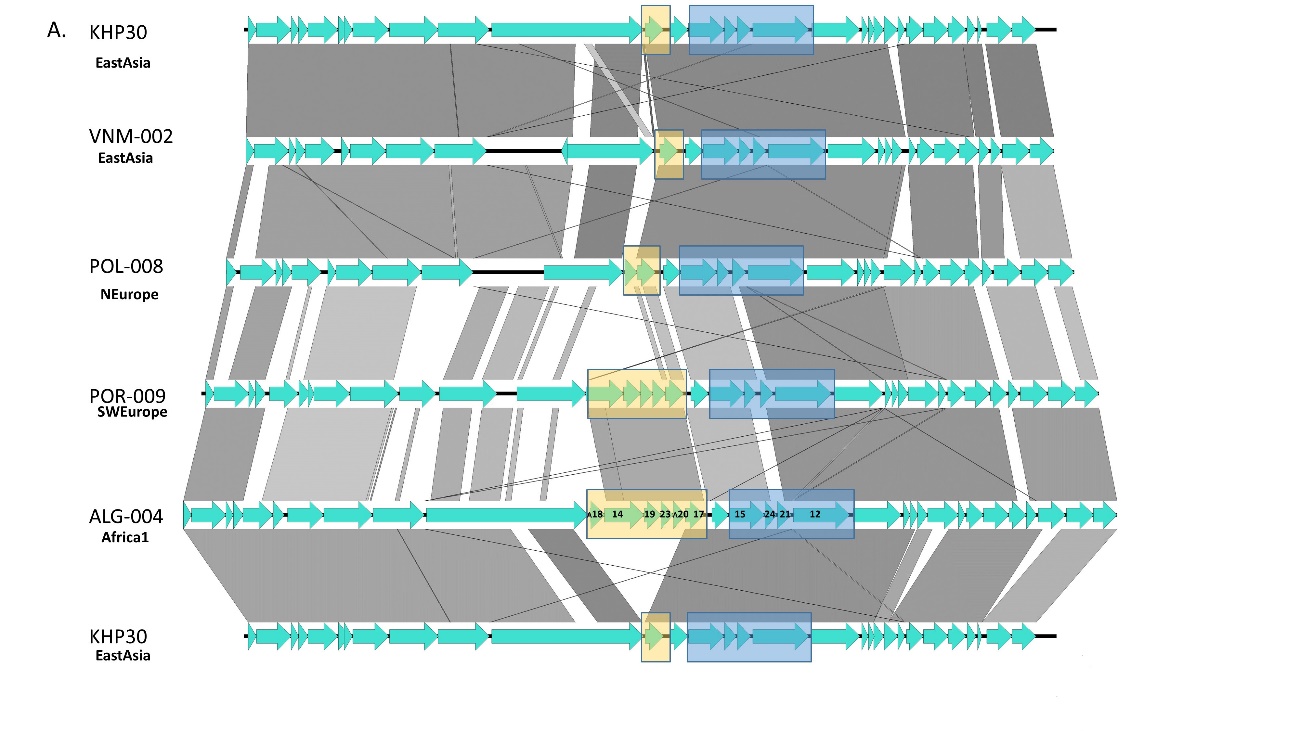


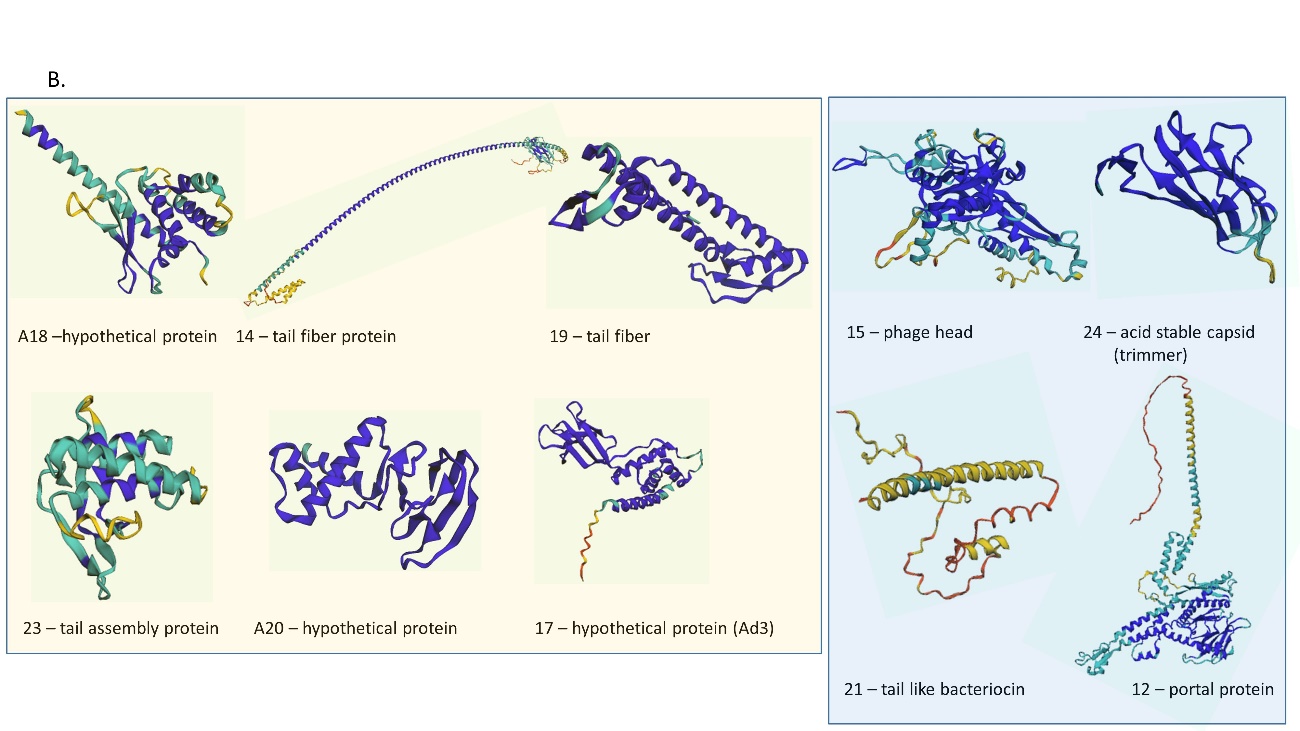


**Figure S5**. **A**. Gene cluster evidencing structural coding genes for the reference 4 prophage. **B**. Prediction of protein structure using Alphafold2.

# **Supplementary Tables**

**Table S1**. *H. pylori* prophage distribution by country of isolation.

| \| **Country** \| **Total number of Genomes** \| **Genomes with prophage (n)** \| **Genomes with prophages (%)** \| **Genomes with complete prophages (n)** \| **Genomes with complete prophages (%)** \| **Proportion of complete prophages (%)** \| \| --- \| --- \| --- \| --- \| --- \| --- \| --- \| \| Algeria \| 10 \| 4 \| 40 \| 3 \| 30 \| 75 \| \| Argentina \| 10 \| 4 \| 40 \| 2 \| 20 \| 50 \| \| Bangladesh \| 10 \| 2 \| 20 \| 1 \| 10 \| 50 \| \| Brazil \| 21 \| 8 \| 38.1 \| 5 \| 24 \| 63 \| \| Bulgaria \| 8 \| 3 \| 37 \| 1 \| 13 \| 33 \| \| Canada \| 20 \| 0 \| 0.0 \| 0 \| 0 \|  \| \| Chile \| 46 \| 15 \| 32.6 \| 3 \| 7 \| 20 \| \| China \| 10 \| 4 \| 40 \| 3 \| 30 \| 75 \| \| Colombia \| 45 \| 8 \| 17.8 \| 2 \| 4 \| 25 \| \| Costa Rica \| 8 \| 4 \| 50 \| 2 \| 25 \| 50 \| \| Dominican Republic \| 11 \| 6 \| 54.6 \| 2 \| 18 \| 33 \| \| DR Congo \| 11 \| 1 \| 9 \| 1 \| 9 \| 100 \| \| France \| 21 \| 4 \| 19 \| 2 \| 10 \| 50 \| \| Gambia \| 5 \| 0 \| 0 \| 0 \| 0 \|  \| \| Germany \| 17 \| 7 \| 41.2 \| 4 \| 24 \| 57 \| \| Ghana \| 2 \| 0 \| 0 \| 0 \| 0 \|  \| \| Greece \| 21 \| 8 \| 38.1 \| 6 \| 28.6 \| 75.0 \| \| Guatemala \| 3 \| 0 \| 0 \| 0 \| 0 \|  \| \| Honduras \| 26 \| 3 \| 11.5 \| 0 \| 0 \| 0 \| \| Iceland \| 11 \| 1 \| 9 \| 0 \| 0 \| 0 \| \| India \| 10 \| 3 \| 30 \| 2 \| 20 \| 67 \| \| Indonesia \| 11 \| 2 \| 18 \| 1 \| 9 \| 50 \| \| Iran \| 4 \| 1 \| 25 \| 0 \| 0 \| 0 \| \| Israel \| 10 \| 4 \| 40 \| 2 \| 20 \| 50 \| \| Italia \| 29 \| 8 \| 27.6 \| 0 \| 0 \| 0 \| \| Japan \| 29 \| 1 \| 4 \| 0 \| 0 \| 0 \| \| Jordan \| 10 \| 6 \| 60.0 \| 2 \| 20 \| 33 \| \| Kazakhstan \| 2 \| 0 \| 0 \| 0 \| 0 \|  \| \| Korea \| 54 \| 11 \| 20.4 \| 2 \| 4 \| 18 \| \| Kyrgyzstan \| 10 \| 4 \| 40 \| 2 \| 20 \| 50 \| \| Latvia \| 34 \| 19 \| 55.9 \| 6 \| 17.7 \| 31.6 \| \| Lithuania \| 23 \| 9 \| 39.1 \| 3 \| 13 \| 33 \| \| Malaysia \| 19 \| 4 \| 21 \| 2 \| 11 \| 50 \| \| Mexico \| 22 \| 2 \| 9 \| 1 \| 5 \| 50 \| \| Myanmar \| 12 \| 1 \| 8 \| 0 \| 0 \| 0 \| \| Nepal \| 13 \| 3 \| 23 \| 1 \| 8 \| 33 \| \| Nigeria \| 4 \| 2 \| 50 \| 0 \| 0 \| 0 \| \| Peru \| 33 \| 8 \| 24.2 \| 1 \| 3 \| 13 \| \| Poland \| 20 \| 11 \| 55.0 \| 6 \| 30.0 \| 54.6 \| \| Portugal \| 30 \| 11 \| 36.7 \| 3 \| 10 \| 27 \| \| Puerto Rico \| 7 \| 1 \| 14 \| 1 \| 14 \| 100 \| \| Russia \| 10 \| 7 \| 70.0 \| 1 \| 10 \| 14 \| \| Singapore \| 21 \| 6 \| 28.6 \| 2 \| 10 \| 33 \| \| South Africa \| 9 \| 3 \| 33 \| 2 \| 22 \| 66 \| \| Spain \| 106 \| 39 \| 36.8 \| 11 \| 10.4 \| 28.2 \| \| Sweden \| 30 \| 8 \| 26.7 \| 0 \| 0 \| 0 \| \| Switzerland \| 15 \| 1 \| 7 \| 0 \| 0 \| 0 \| \| Taiwan \| 24 \| 2 \| 8 \| 1 \| 4 \| 50 \| \| Turkey \| 17 \| 2 \| 12 \| 1 \| 6 \| 50 \| \| USA \| 68 \| 34 \| 50.0 \| 3 \| 4 \| 9 \| \| Vietnam \| 9 \| 3 \| 33 \| 3 \| 33 \| 100 \| \| **Total** \| **1011** \| **298** \| **29.5** \| **96** \| **32.2** \| **32.2** \| |  |  |  |  |  |  |
| --- | --- | --- | --- | --- | --- | --- | --- | --- | --- | --- | --- | --- | --- | --- | --- | --- | --- | --- | --- | --- | --- | --- | --- | --- | --- | --- | --- | --- | --- | --- | --- | --- | --- | --- | --- | --- | --- | --- | --- | --- | --- | --- | --- | --- | --- | --- | --- | --- | --- | --- | --- | --- | --- | --- | --- | --- | --- | --- | --- | --- | --- | --- | --- | --- | --- | --- | --- | --- | --- | --- | --- | --- | --- | --- | --- | --- | --- | --- | --- | --- | --- | --- | --- | --- | --- | --- | --- | --- | --- | --- | --- | --- | --- | --- | --- | --- | --- | --- | --- | --- | --- | --- | --- | --- | --- | --- | --- | --- | --- | --- | --- | --- | --- | --- | --- | --- | --- | --- | --- | --- | --- | --- | --- | --- | --- | --- | --- | --- | --- | --- | --- | --- | --- | --- | --- | --- | --- | --- | --- | --- | --- | --- | --- | --- | --- | --- | --- | --- | --- | --- | --- | --- | --- | --- | --- | --- | --- | --- | --- | --- | --- | --- | --- | --- | --- | --- | --- | --- | --- | --- | --- | --- | --- | --- | --- | --- | --- | --- | --- | --- | --- | --- | --- | --- | --- | --- | --- | --- | --- | --- | --- | --- | --- | --- | --- | --- | --- | --- | --- | --- | --- | --- | --- | --- | --- | --- | --- | --- | --- | --- | --- | --- | --- | --- | --- | --- | --- | --- | --- | --- | --- | --- | --- | --- | --- | --- | --- | --- | --- | --- | --- | --- | --- | --- | --- | --- | --- | --- | --- | --- | --- | --- | --- | --- | --- | --- | --- | --- | --- | --- | --- | --- | --- | --- | --- | --- | --- | --- | --- | --- | --- | --- | --- | --- | --- | --- | --- | --- | --- | --- | --- | --- | --- | --- | --- | --- | --- | --- | --- | --- | --- | --- | --- | --- | --- | --- | --- | --- | --- | --- | --- | --- | --- | --- | --- | --- | --- | --- | --- | --- | --- | --- | --- | --- | --- | --- | --- | --- | --- | --- | --- | --- | --- | --- | --- | --- | --- | --- | --- | --- | --- | --- | --- | --- | --- | --- | --- | --- | --- | --- | --- | --- | --- | --- | --- | --- | --- | --- | --- | --- | --- | --- | --- | --- | --- | --- | --- | --- | --- | --- | --- | --- | --- | --- | --- | --- | --- | --- | --- | --- | --- | --- | --- | --- | --- | --- | --- | --- | --- | --- | --- | --- | --- | --- | --- | --- | --- |
| **Table S2**. Detailed identification of prophages and their insertion site at bacterial genome. Excel file. |  |  |  |  |  |  |

| **Table S3**. Prophage insertion site at restriction and modification genes: impact on gene disruption and DNA methylation at recognition site.  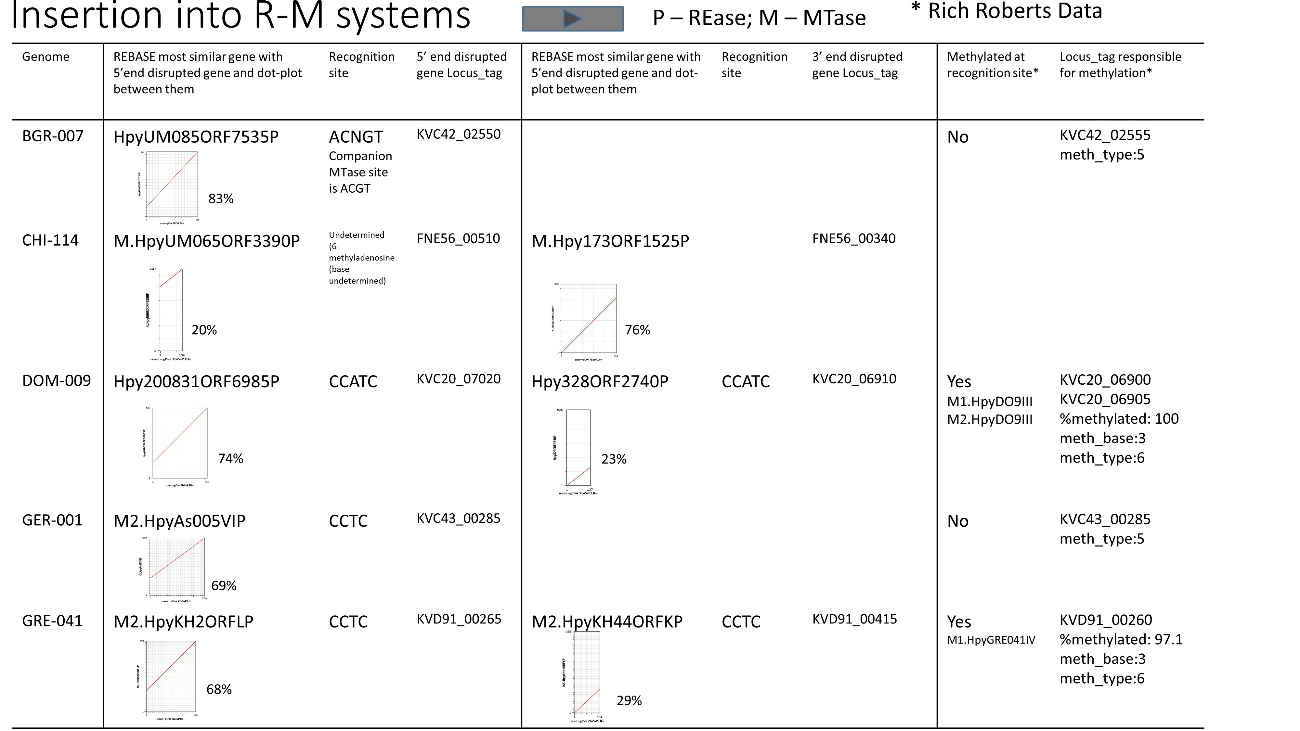  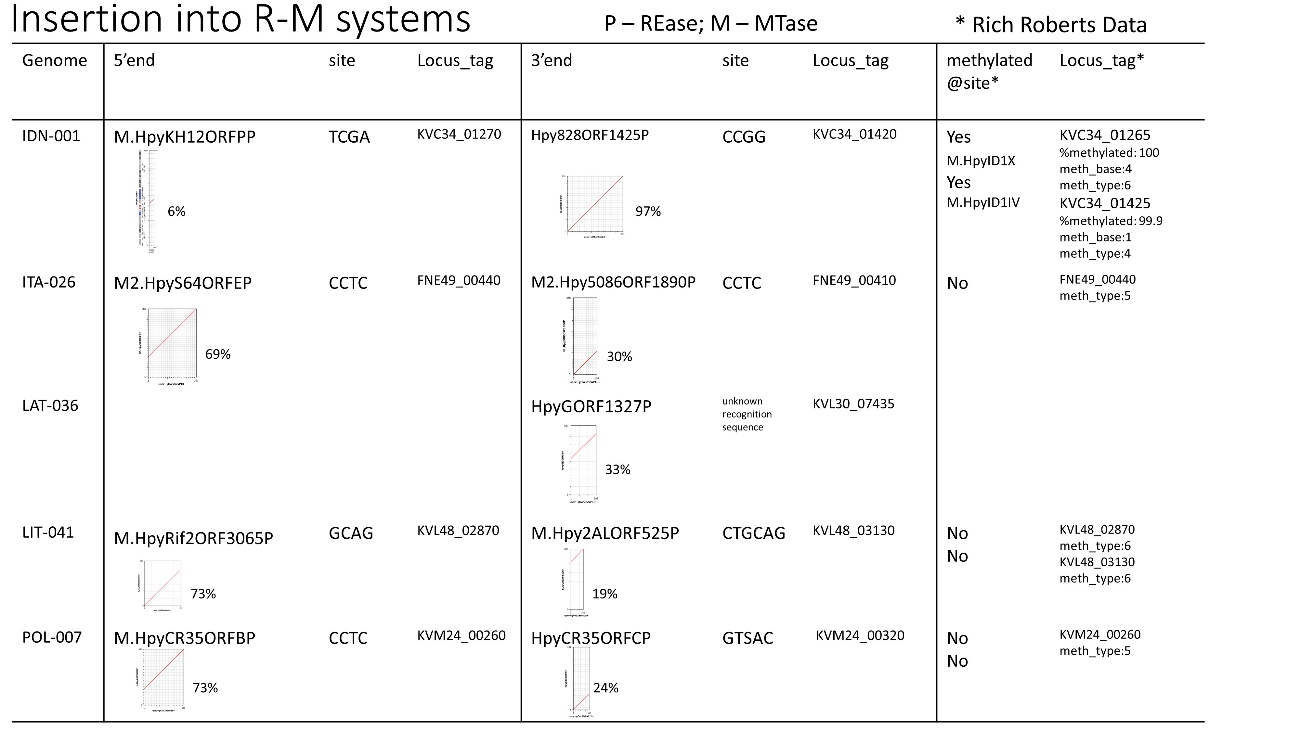  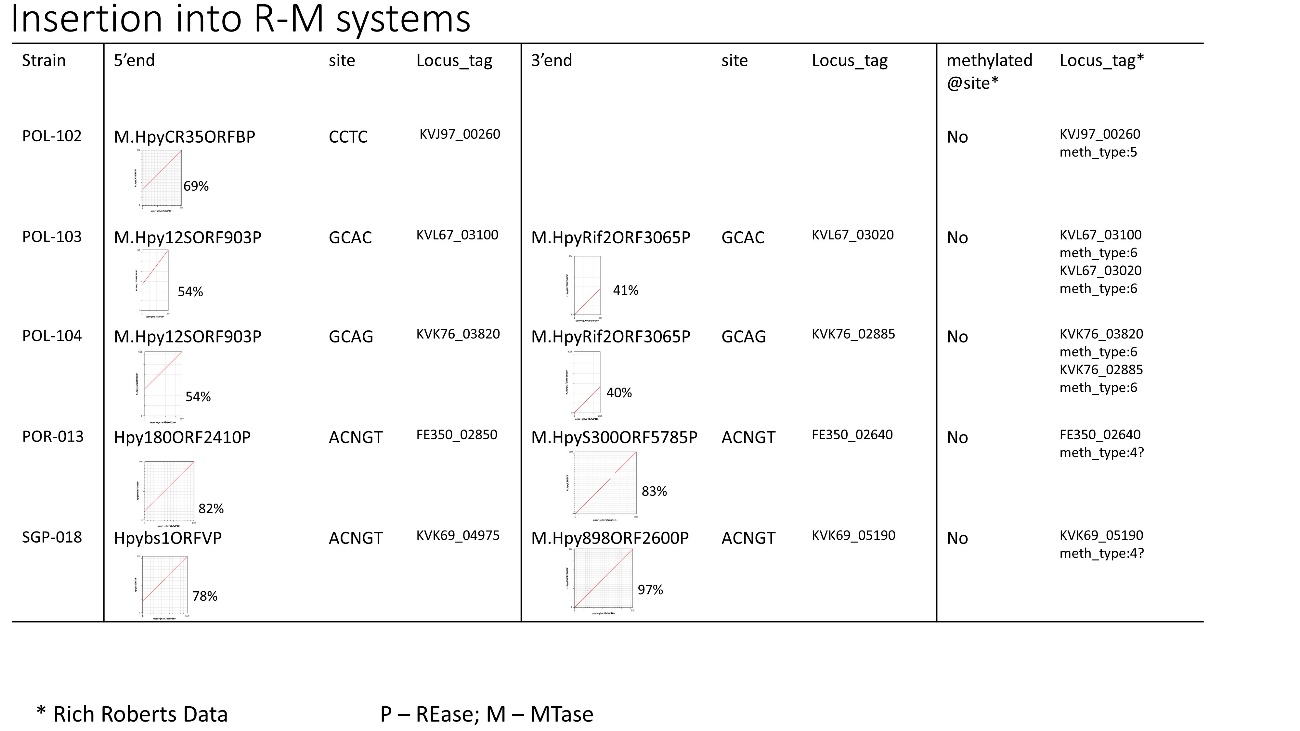  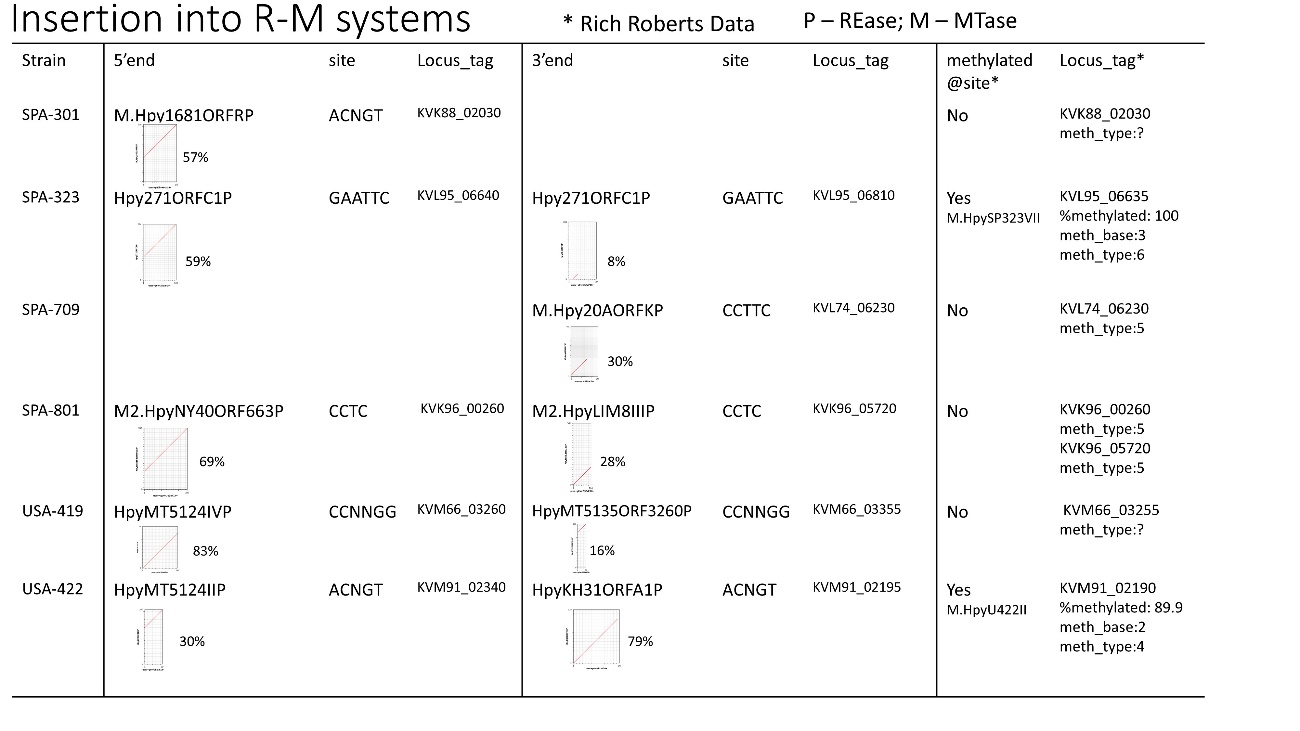  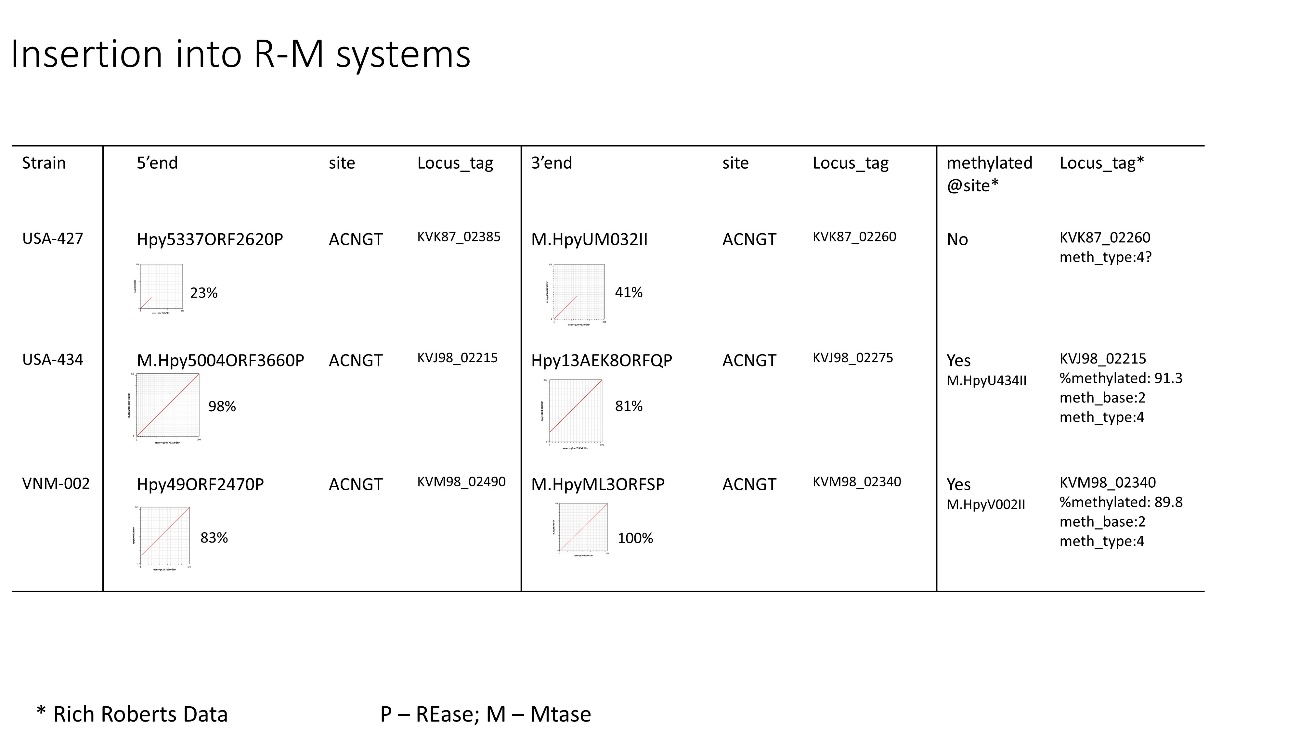  * PACBIO determination of methylation status at recognition site and identification of locus tag coding for the methyltransferase that methylates the recognition site. 62.5% (15/24) of the prophage genomes flanked by RM genes present RM disruption, disrupting bacterial genomic methylation. Meth_type: methylation type: 4 - N4-methylcytosine; 5 - C5-methylcytosine;6 - N6-adenine. The question mark means that the methylation type is not certain). Meth_base: position of the methylated base in the recognition site.  Note: The locus tag of the restriction or modification gene flaking the prophage is given; noting that if the prophage insertion is closer to the end of the gene the smallest part of the disrupted gene may not have been annotated (for instance, in the case of BGR-007). The columns named 5’end and 3’end show the most similar gene name found at REBASE, and the dot-plot between that gene and the gene disrupted by the prophage (for instance for BGR-007, the figure shows the dot-plot between the REBASE gene HpyUM0850RF7535P and the disrupted gene with locus tag KVC42_02550). The identity percentage between disrupted gene and REBASE gene is presented. Frequently the sum of the identity of the gene disrupted at 5’end and at 3’end is near 100%, meaning that part of the disrupted gene is at 5’end of the prophage and the rest of the gene is at 3’end of the prophage. For instance, in the case of BGR-007, the disrupted locus tag was KVC42_02550, which codes for a restriction endonuclease (the most similar genes in REBASE do not start with an 'M'), and its companion methyltransferase is associated with the locus tag KVC42_0255, whose recognition site is ACGT and has not been found to be methylated. |  |  |  |  |  |  |
| --- | --- | --- | --- | --- | --- | --- |

| **Table S4**. Disrupted genes at insertion site.   \| **Gene** \| **Number** \| **GO TERMS** \| \| --- \| --- \| --- \| \| Hypothetical protein \| 40 \|  \| \| MTase \| 22 \| Transferase activity> Catalytic activity> Molecular Function  Methylation > metabolic process > biological process \| \| REase \| 10 \| Hydrolase activity > Catalytic activity > Molecular Function  Nitrogen compound metabolic > metabolic process > biological process \| \| Outer membrane protein \| 8 \| Membrane protein complex> protein-containing complex> celular component \| \| ATP-binding protein \| 3 \| Protein binding > binding > Molecular function \| \| sel1 repeat family protein \| 3 \| Protein binding > binding > Molecular function \| \| type IV secretory system conjugative DNA transfer family protein \| 3 \| type IV secretion system complex > Protein containing complex > celular component \| \|  \|  \| Export from cell > celular process > biological process \| \| DNA topoisomerase \| 2 \| Isomerase > Catalytic activity > Molecular Function \| \| transaldolase \| 2 \| Transferase > Catalytic activity> Molecular Function \| \| TrbG/VirB9 family P-type conjugative transfer protein \| 2 \| establishment of localization > Localization > biological process \| \| AAA family ATPase \| 1 \| Hydrolase activity > Catalytic activity > Molecular Function  Catalylic complex > protein containing complex > celular component \| \| ABC transporter ATP-binding protein \| 1 \| Transporter complex > Protein containing complex > celular component  transmembrane transporter activity > Transporter activity > Molecular Function \| \| DEAD/DEAH box helicase family protein \| 1 \| Protein binding > binding > Molecular function \| \| DNA-binding protein \| 1 \| Heterocyclic compound binding > binding > Molecular function \| \| DUF1542 \| 1 \|  \| \| DUF3519 \| 1 \|  \| \| Helicase \| 1 \| Catalytic activity, acting on a nucleic acid > Catalytic activity> Molecular Function \| \| lpxD\|UDP-3-O-(3-hydroxymyristoyl)glucosamine N-acyltransferase \| 1 \| Transferase activity> Catalytic activity> Molecular Function \| \| metK\|methionine adenosyltransferase \| 1 \| Transferase activity> Catalytic activity> Molecular Function \| \| Relaxase \| 1 \|  \| \| S-adenosyl-l-methionine hydroxide adenosyltransferase family protein \| 1 \| Transferase activity> Catalytic activity> Molecular Function \| \| Transporter \| 1 \| Transporter activity > Molecular Function \| \| Transposase \| 1 \| Catalytic activity, acting on a nucleic > Catalytic activity> Molecular Function \| \| Virulence factor \| 1 \|  \|   Note: 14.1% (109/(96+272)*2) of the prophage elements generate bacterial gene fragments at insertion site either/both at 5’end or/and 3’end.  **Table S5**. Genomic islands (mobilome) convergence in bacterial genome. Merging of *H. pylori* prophages with other mobile elements.   \| **Mobile element** \| **Number** \| **% (n = 298)** \| **Observation** \| \| --- \| --- \| --- \| --- \| \| Insertion sequence \| 145 \| 48.0 \| IS605, IS606 or IS607 \| \| Type IV secretion (*tfs*) cluster \| 34 \| 10.4 \| Prophage insertion flanking Tfs3 c10, c13 or c21 genes;  Tfs4 *xerT* or c19 genes \| \| Adjacent to or split *tfs* \| 14 \| 4.7 \|  \| \| Within *tfs* \| 9 \| 3.2 \|  \| \| Cargo *tfs* genes \| 11 \| 3.7 \|  \| \| *cag* pathogenicity island (PAI) \| 10 \| 3.4 \| Prophage insertion flanking *cag4* or *cagS* genes \| \| Adjacent to or split *cag*PAI \| 6 \| 2.0 \|  \| \| Within *cag*PAI \| 4 \| 1 \|  \|   Note: Contribution of phages for disruption of *tfs* and *cag*PAI and vice-versa.  **Table S6**. Predicted gene function of the prophage pangenome (coding 147 proteins). Excel file. |  |  |  |  |  |  |
| --- | --- | --- | --- | --- | --- | --- | --- | --- | --- | --- | --- | --- | --- | --- | --- | --- | --- | --- | --- | --- | --- | --- | --- | --- | --- | --- | --- | --- | --- | --- | --- | --- | --- | --- | --- | --- | --- | --- | --- | --- | --- | --- | --- | --- | --- | --- | --- | --- | --- | --- | --- | --- | --- | --- | --- | --- | --- | --- | --- | --- | --- | --- | --- | --- | --- | --- | --- | --- | --- | --- | --- | --- | --- | --- | --- | --- | --- | --- | --- | --- | --- | --- | --- | --- | --- | --- | --- | --- | --- | --- | --- | --- | --- | --- | --- | --- | --- | --- | --- | --- | --- | --- | --- | --- | --- | --- | --- | --- | --- | --- | --- | --- | --- | --- | --- | --- | --- | --- | --- | --- |
| **Table S7**. *H. pylori* prophage cargo genes.   \|  \| **All prophages** \| \| **Complete prophages** \| \|  \| \| --- \| --- \| --- \| --- \| --- \| --- \| \| **Cargo gene** \| **Number** \| **% (n = 298)** \| **Number** \| **% (n = 96)** \| **Observation** \| \| *tfs* gene \| 12 \| 4.0 \| 2 \| 2 \| Intermediate virulence phenotype \| \| Toxin-antitoxin \| 11 \| 3.7 \| 4 \| 4 \| Prophage addition, post-segregational killing effect \| \| Bacterial gene block  *metK*  *ndk*  hypothetical protein  *rpmF*  *plsX*  ketoacyl-ACP synthase III \| 1 \| 0.3 \| 1 \| 1 \| Prophage genome inversion, involving nearby bacterial genes \| \| DNA N-6 methyltransferase \| 1 \| 0.3 \| 1 \| 1 \| Unknown methylation site \| \| Total \| 25 \| 10.4 \| 8 \| 8.3 \|  \| |  |  |  |  |  |  |
|  |  |  |  |  |  |  |
